# One-dimensional inorganic ionic polymerization for elastic minerals

**DOI:** 10.1038/s41467-026-72767-0

**Published:** 2026-05-27

**Authors:** Yongjin Du, Zeyu Gong, Ruoyan He, Manfang Hu, Lina Zhou, Wenge Jiang, Yadong Yu, Junbo Gong

**Affiliations:** 1 School of Chemical Engineering and Technology, Tianjin University, Tianjin, China; 2 Department of Chemistry, Tianjin University, Tianjin, China

**Keywords:** Mechanical properties, Organic-inorganic nanostructures

## Abstract

The inherent brittleness arising from atomic close-packing of inorganic ionic minerals significantly limits their potential applications as elastic materials. Inspired by elastomers and tough minerals, we propose a one-dimensional (1D) inorganic ionic polymerization tactic for developing elastic minerals. Guided by polyvinyl alcohol (PVA) chains, calcium silicate oligomers (CSO) undergo 1D polymerization, forming highly flexible PVA/CSO ionic-molecular chains. These chains are hierarchically assembled into nanofibers and bundles, which further crosslink to a flexible network. This structure enables the PVA/CSO bulk to maintain high hardness (~0.78 GPa), Young’s modulus (~20.63 GPa), and specific strength (~74.53 MPa g⁻¹ cm³), while also achieving elastic recovery after 5000 cycles at 10% strain, hence being termed an elastic mineral. Furthermore, smart elastic minerals have been developed and serve as sensors for real-time warning. The proposed 1D inorganic ionic polymerization tactic breaks the boundaries between minerals and elastomers, paving the way for producing high-performance elastic minerals.

## Abstract (web summary)

Through a one-dimensional inorganic ionic polymerization strategy, researchers have successfully obtained elastic minerals that combine high hardness with elastomer-like flexibility, which hold promise for high-strength intelligent strain sensors.

## Introduction

Inorganic ionic minerals (such as calcium carbonate^1,2^, calcium phosphate^3^, and calcium silicate (CaS)^4,5^), as natural materials widely present in the Earth’s crust, play a crucial role in numerous fields including construction^6^, optics^7^, biomedicine^8,9^, and structural engineering^10–12^ due to their high hardness and strength, thereby driving the progress of human society. However, the strong electrostatic interactions between ions in these materials facilitate the spontaneous arrangement of ions into regular and dense rigid crystal lattices. During irreversible deformation induced by external stress, relative slip between ion layers can bring like-charged ions into close proximity, initiating strong electrostatic repulsion. This repulsion quickly overcomes the original electrostatic attraction, promoting rapid crack propagation along cleavage planes and ultimately resulting in the material’s brittle fracture. In contrast, elastomers, such as natural rubber and synthetic silicone rubber^13–15^, consist of crosslinked long-chain molecules forming a three-dimensional (3D) polymer network, with high elasticity arising from an entropy-driven recovery mechanism^16^. During macroscopic deformation, molecular chains shift from a disordered coiled state to an aligned orientation, leading to a notable decrease in conformational entropy. Upon removal of the external stress, the system spontaneously returns to a high-entropy state driven by thermal motion, thereby enabling elastic recovery of large deformations. Thus, a fundamental distinction exists in the microscopic structures and energy-response deformation mechanisms of inorganic ionic minerals versus polymer elastomers, which renders it challenging for inorganic ionic minerals to display macroscopic elastic behavior comparable to that of elastomers. Consequently, their intrinsic brittleness significantly restricts their applicability in tough structural materials, particularly in applications requiring elasticity. Designing an energy-dissipation network analogous to that of elastomers at the microscopic scale, while preserving the intrinsic properties of the inorganic phase to confer recoverable deformation on inorganic ionic crystals, remains challenging.

In recent years, studies indicate that the crystallization pathways of inorganic ions can be modulated by specific organic molecules, facilitating advances in their elastic mechanical properties^17–19^. Specifically, polymers exhibiting strong specific interactions, such as electrostatic interactions or hydrogen bonding with inorganic crystal precursors, are employed to suppress disordered nucleation and ion aggregation, thereby maintaining the colloidal stability of nanoscale building units. Subsequently, these nanoscale units undergo guided ordered self-assembly, yielding organic–inorganic hybrid materials with mesocrystalline structures. These mesocrystals comprise highly ordered inorganic nanocrystals embedded within a continuous organic matrix. The inorganic phase provides mechanical strength, whereas the organic phase functions as an energy-dissipating medium, conferring toughness and enabling substantial elastic deformation and recovery at the mesoscopic scale, thus overcoming the intrinsic brittleness of conventional ionic crystals^19^. However, due to the challenges in constructing mesocrystalline structures at the macroscopic scale, this strategy currently achieves plastic deformation and elastic recovery primarily at the nanoscale to microscale, with performance confined to discrete mesoscopic units and not yet effectively extended to bulk material systems.

Inorganic ionic oligomers, a class of precursors suitable for the moldable construction of macroscopic inorganic ionic minerals^20^, have recently been found to form flexible inorganic mineral nanofibers containing periodic structural defects through inorganic ionic polymerization induced by organic molecules^21,22^. Upon crosslinking, these nanofibers can yield tough minerals exhibiting limited plastic deformation capabilities. However, owing to the short lengths of these nanofibers (typically < 200 nm), the limited number of internal defects, and low curvature, the inorganic network formed by crosslinked short nanofibers struggles to achieve effective macroscopic elastic recovery following plastic deformation. Nonetheless, the strategy of constructing tough minerals via inorganic ionic polymerization suggests that by further employing organic molecules to regulate inorganic ionic polymerization, forming longer one-dimensional (1D) flexible inorganic ionic chains with increased ionic defects to enhance deformability, and subsequently crosslinking these long chains into a 3D continuous network, could potentially produce elastic minerals exhibiting substantially improved toughness and polymer-like elastomeric behavior.

Inspired by the above findings, herein, we propose a 1D inorganic ionic polymerization tactic for constructing elastic minerals. Specifically, CaS (a typical engineering mineral) oligomers (CSO) are employed as precursors, and 1D inorganic ionic polymerization is induced by linear polyvinyl alcohol (PVA) chains rich in hydroxyl groups, initially forming highly curved and flexible PVA/CSO composite ionic-molecular chains. These ionic-molecular chains hierarchically assemble into PVA/CSO nanofibers and bundles, ultimately crosslinking to form a 3D continuous network, facilitating the construction of macroscopic PVA/CSO bulk. The resulting PVA/CSO bulk, containing 58.1 wt% CaS, exhibits a 3D network structure analogous to that of polymer elastomers. This distinctive structure enables the PVA/CSO bulk to retain the high hardness (0.78 ± 0.11 GPa), Young’s modulus (20.63 ± 1.77 GPa), and specific strength (74.53 ± 3.56 MPa g⁻¹ cm³) characteristic of inorganic ionic minerals while overcoming inherent brittleness, exhibiting plastic deformation with compressive strains exceeding 50% and achieving complete structural recovery within a 10% compressive strain. Given its mineral-based composition and exceptional deformation and elastic properties, it is designated as PVA/CSO elastic mineral. Moreover, after 5000 compressive cycles at 10% strain, the PVA/CSO elastic mineral maintains its original structure and mechanical properties, demonstrating wonderful fatigue resistance. Additionally, through the incorporation of functional nanomaterials, such as conductive graphite, into the construction process of PVA/CSO elastic minerals, we have developed a smart PVA/CSO/Graphite (PCG) elastic mineral capable of functioning as a strain sensor. The PCG elastic mineral can be applied in dynamic loading environments, such as those encountered during earthquakes and bridge overloads, for real-time early warning, thereby demonstrating significant potential for applications in structural engineering. The proposed 1D inorganic ionic polymerization tactic not only advances the understanding of the concept and structural design of elastic minerals but also breaks the boundaries between traditional inorganic ionic minerals and polymer elastomers, thus paving the way for the development of novel high-performance elastic mineral materials.

## Results

### 1D inorganic ionic polymerization of CSO

**Fig. 1:**
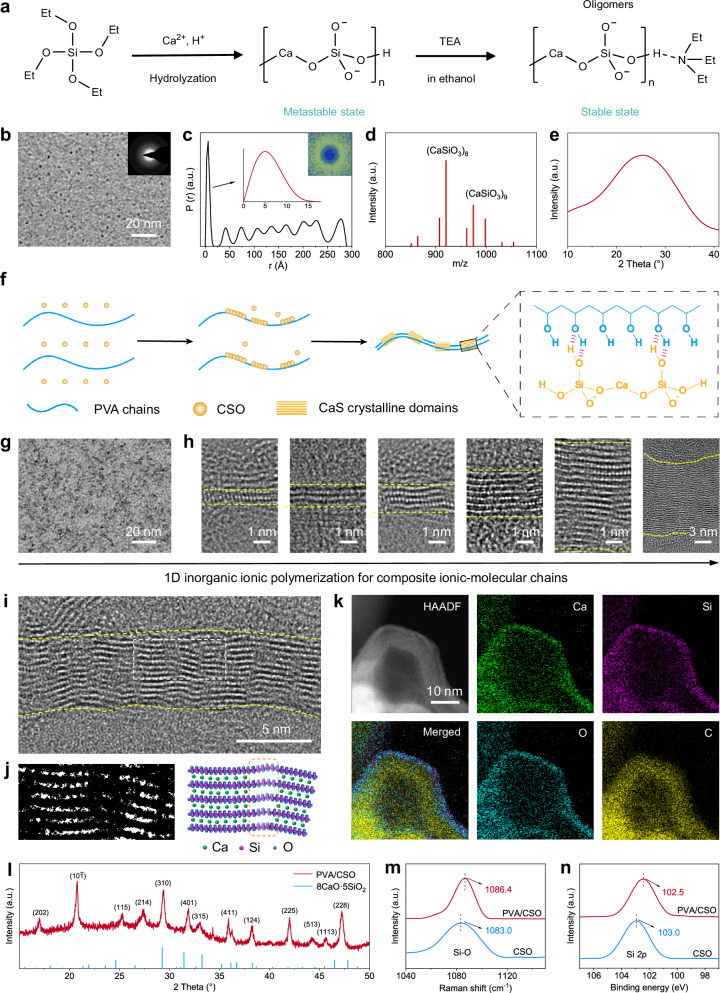
1D inorganic ionic polymerization of CSO. **a** Schematic illustration of the synthesis process of CSO using TEA molecules as capping agents and ethyl orthosilicate hydrolysis. **b** TEM image of CSO, with the inset showing the corresponding SAED pattern. **c** Pair-distance distribution function P(r) of the CSO. The inset displays the 2D SAXS pattern of the CSO dispersion. **d** MALDI-TOF mass spectrum of CSO at a Ca²⁺ to TEA molar ratio of 1:20. **e** XRD pattern of dried CSO. **f** Schematic illustration of the synthesis process of the flexible PVA/CSO ionic-molecular chains and nanofibers via 1D inorganic ionic polymerization of CSO, induced by linear PVA chains. **g** TEM image of the PVA/CSO precursor slurry at the initial state. The sample was prepared by rapidly mixing CSO gel and PVA solution to form a uniform slurry for 1 min, followed by TEM observation. **h** Cs-corrected TEM images of PVA/CSO nanofibers with varying diameters, assembled by different numbers of PVA/CSO ionic-molecular chains. **i** Cs-corrected TEM image of the PVA/CSO nanofiber. **j** Binary image and schematic diagram of the relative atomic arrangement within the white dashed box in panel (**i**). **k** HAADF-STEM micrograph of PVA/CSO nanofibers, with the corresponding elemental mapping of Ca, Si, O, and C. **l** XRD pattern of PVA/CSO nanofibers. **m** Raman spectra of PVA/CSO nanofibers and CSO. **n** High-resolution XPS spectra of Si 2p in PVA/CSO nanofibers and CSO.

Tough minerals, composed of crosslinked networks of calcium phosphate nanofibers, have been produced by controlling the inorganic polymerization of calcium phosphate oligomers in previous studies^21^. However, owing to the limited internal defects and bending curvature of the calcium phosphate nanofibers serving as building blocks, the material exhibited restricted plastic deformation at the macroscopic scale. This behavior originates from the strong electrostatic interactions between phosphate and calcium ions during the polymerization of calcium phosphate oligomers, which promote rapid bonding through ionic interactions, leading to ionic defects and flexible crystalline nanofibers of limited length. To construct elastic minerals, we propose introducing additional ionic defects into the mineral nanofibers, thereby producing longer and more curved flexible nanofibers. Based on this concept, CSO was selected as the inorganic precursor for elastic minerals (Fig. 1a). Compared with phosphate ions, silicate ions exhibit weaker binding with calcium ions, facilitating the formation of flexible crystalline nanofibers enriched with ionic defects and possessing higher curvature. Specifically, the hydrolysis of tetraethyl orthosilicate (TEOS) was carefully controlled to release silicate ions, which subsequently reacted with calcium ions in ethanol. Triethylamine (TEA) was employed as a capping agent, with the calcium-to-TEA molar ratio fixed at 1:20, to synthesize CSO. This ratio ensures effective surface capping while preventing particle enlargement caused by insufficient TEA and minimizing undesirable side reactions induced by excess TEA. The dilute CSO dispersion exhibited a pronounced Tyndall effect, indicating its excellent dispersibility and suspension stability in ethanol (Supplementary Fig. 1a). Furthermore, the synthesis was carried out in an open system at 25 °C without requiring special conditions, thereby facilitating large-scale preparation of CSO and laying the foundation for its subsequent scaled-up applications. Transmission electron microscopy (TEM) image revealed that CSO forms approximately spherical, uniformly dispersed clusters with an average diameter of 1.5 ± 0.3 nm (Fig. 1b and Supplementary Fig. 1b). Selected area electron diffraction (SAED) patterns exhibited no discernible diffraction spots or rings, confirming the amorphous nature of CSO (inset in Fig. 1b).

Subsequently, a detailed investigation into the capping effect of TEA was performed. Optical photograph revealed that the CSO dispersion formed a transparent gel after centrifugation at 5778 × *g* for 5 min (Supplementary Fig. 1c), indicating strong intermolecular interactions between CaS ions and either ethanol or TEA, thereby facilitating the efficient solvation of CaS ions. Concurrently, ethanol as a solvent significantly slows down the hydrolysis and condensation rates of silicates, effectively inhibiting the condensation of silicate ions. The attenuated total reflection–Fourier transform infrared (ATR–FTIR) spectrum revealed that the C–N stretching vibration peak of TEA at 1200 cm⁻¹ shifted to 1203 cm⁻¹ following CSO formation (Supplementary Fig. 1d)^23^. A similar phenomenon was observed in the Raman spectrum, with the C–N stretching vibration peak of TEA shifting from 735.5 cm⁻¹ to 739.0 cm⁻¹ (Supplementary Fig. 1e). These shifts are attributed to the formation of O–H⋯N hydrogen bonds in CSO, which alter the C–N bond vibrational frequency, thereby confirming the role of TEA as a capping agent. This hydrogen bonding interaction not only reduces the condensation reactivity of silanol groups (Si–OH) but also forms a charge protection layer on the surface of CSO, enhancing the repulsive barrier between CSO particles, thereby kinetically hindering their collision, aggregation, and further condensation.

Furthermore, ^29^Si liquid-state nuclear magnetic resonance (NMR) spectroscopy confirmed the interaction between TEA and silicon species. The results demonstrate that the characteristic Si–O bond chemical shift in TEOS shifted from –113.5 ppm^24,25^, through that of the hydrolysis intermediate (–112.5 ppm), to –111.8 ppm in CSO (Supplementary Fig. 1f). This continuous shift reflects the gradual alteration of the silicon atom’s chemical environment resulting from coordination with TEA, further indicating that TEA stabilizes the CSO structure through hydrogen bonding.

1D small-angle X-ray scattering (SAXS) was further employed to analyze the size and morphology of CSO (Fig. 1c). The pair-distance distribution function P(r) showed a sharp first peak at approximately 1.5 nm, indicating the presence of highly uniform, well-defined basic units at the microscopic scale with a characteristic size of roughly 1.5 nm. The subsequent damped oscillatory signal over a range of approximately 28 nm revealed that CSO possesses a long-range ordered structure, suggesting that these primary structural units are capable of spontaneously assembling to form periodic mesophases. This mesoscopic order provides the structural basis for subsequent 1D inorganic ionic polymerization. Additionally, the two-dimensional SAXS image exhibited uniform diffraction ring, further confirming that CSO is isotropic on the macroscopic scale and lacks significant preferential orientation (inset in Fig. 1c). Matrix-assisted laser desorption/ionization time-of-flight (MALDI-TOF) mass spectrometry was employed to determine the molecular weight of CSO (Fig. 1d). Prominent negative ion peaks appeared at m/z 921 and 998 in the spectrum, corresponding to the (CaSiO₃)₈ and (CaSiO₃)₉ species prior to ionization, respectively, indicating that the structural repeat unit of CSO comprises approximately 8 to 9 CaSiO₃ units. The X-ray diffraction (XRD) pattern showed only a broad diffuse peak near 2θ = 25°, with no sharp crystalline diffraction signals, further confirming the amorphous structural characteristics of CSO (Fig. 1e). Furthermore, the as-prepared CSO dispersion, after static storage for approximately 6 months, still exhibited excellent uniform suspendability and fluidity, maintaining an appearance consistent with that of freshly prepared CSO dispersion, without any sedimentation, aggregation, or phase separation (Supplementary Fig. 2a–c). Its XRD pattern remained amorphous (Supplementary Fig. 2d). These results indicate that the CSO synthesized using this strategy possesses good long-term stability.

Similar to previously reported calcium carbonate and calcium phosphate oligomers, CSO can undergo inorganic ionic polymerization during TEA removal, which promotes the growth of CaS ionic chains in 3D space, ultimately forming a continuous and dense structure (Supplementary Fig. 3a–d). This process begins with oligomers, progresses through branched chains and 3D continuous networks, and ultimately yields bulk material, exhibiting a formation mechanism similar to that of certain polymers (Supplementary Fig. 3e). TEM image, SAED pattern, high-angle annular dark-field scanning transmission electron microscopy (HAADF-STEM) image, and corresponding elemental mapping results showed that the formed CaS network is continuous, dense, and amorphous, with Ca, Si, and O uniformly distributed (Supplementary Fig. 3f, g), demonstrating that the inorganic ionic polymerization of CSO in 3D space proceeds freely and without significant directionality. Furthermore, natural drying of the CSO gel yielded CaS bulk material (Supplementary Fig. 3h). Scanning electron microscopy (SEM) images showed that the resulting CaS bulk was continuous and dense yet prone to developing numerous flat cracks, exhibiting the typical brittle characteristics of inorganic ionic minerals (Supplementary Fig. 3i–k).

Inspired by the formation of flexible nanofibers via the inorganic ionic polymerization of calcium phosphate oligomers, we introduced 1D linear polyvinyl alcohol (PVA) molecular chains into the polymerization process of CSO to suppress 3D polymerization and crosslinking, thereby promoting 1D inorganic ionic polymerization (Fig. 1f). This strategy facilitated the combination of CSO with PVA molecular chains to form organic–inorganic composite ionic-molecular chains rather than a 3D ionic network. The PVA molecular chains and CaS ions are connected via O–H⋯O hydrogen bonds, which will be demonstrated later. As shown in Fig. 1g, when CSO is uniformly dispersed within the network of PVA molecular chains, it gradually undergoes 1D polymerization as TEA volatilizes, forming PVA/CSO composite ionic-molecular chains. Spherical aberration-corrected TEM (Cs-corrected TEM) images revealed that these PVA/CSO composite ionic-molecular chains align parallel to one another, assembling into nanofibers with widths ranging from 0.72 to 12.30 nm (Fig. 1h, i). After binarization of the image, the segmented structure of the composite chains and the nanofibers they form could be further identified (Fig. 1j), revealing distinct ionic vacancies at the interruptions. Furthermore, the 1D SAXS pattern confirmed the presence of nanofibers with an average width of 9.1 nm within the PVA/CSO system (Supplementary Fig. 4), which is consistent with the TEM observations. This finding further demonstrates that, facilitated by the PVA molecular chains, CSO undergo oriented polymerization, forming PVA/CSO ionic-molecular chains that self-assemble into nanofibers. Inductively coupled plasma optical emission spectrometry (ICP-OES) revealed that the calcium-to-silicon molar ratio in the nanofibers is 1.25, markedly lower than the value of 1.60 in stoichiometric crystalline CaS (8CaO·5SiO₂, Supplementary Fig. 5a), confirming the presence of numerous Ca^2+^ vacancies. These missing Ca^2+^ ions are randomly distributed between adjacent PVA/CSO ionic-molecular chains, effectively isolating them and thereby imparting notable toughness and elasticity. Statistical analysis of the amorphous gaps within the chain segments showed lengths concentrated around 0.5 nm (Supplementary Fig. 5b), consistent with the size of a single Si–O tetrahedron^26^, suggesting that the local composition of these disordered regions corresponds to a silicate phase. HAADF-STEM image revealed that the PVA/CSO nanofibers exhibit pronounced bending characteristics. The corresponding elemental mapping indicated a uniform distribution of Ca, Si, and O elements derived from the CaS phase, as well as C element originating from the PVA molecular chains (Fig. 1k). Thermogravimetric analysis (TGA) revealed that the mineral content of the PVA/CSO nanofibers is 58.1 wt% (Supplementary Fig. 5c), further confirming their composition of both PVA and CSO. Compared with previously reported calcium phosphate nanofibers, the PVA/CSO nanofibers exhibit a higher density of ionic vacancies and greater bending curvature, which provides the structural basis for their macroscopic elastic behavior (Fig. 1i–k).

XRD pattern further indicated that the phase composition of PVA/CSO nanofibers is generally consistent with that of standard crystalline 8CaO·5SiO₂ (Fig. 1l). However, the diffraction intensities of the (202), (214), and (225) crystal planes are significantly higher. This phenomenon arises from Ca²⁺ vacancies in the PVA/CSO ionic-molecular chains, which alter interchain spacing and disrupt the long-range structural periodicity. Consequently, specific crystal plane families in the CaS phase develop preferential spatial orientation, ultimately enhancing the diffraction signals from these planes. Raman spectroscopy revealed that the characteristic Si–O vibration peak of CSO at 1083.0 cm⁻¹ shifted to 1086.4 cm⁻¹ after forming PVA/CSO (Fig. 1m)^27–29^. X-ray photoelectron spectroscopy (XPS) analysis showed that the Si *2p* binding-energy peak in CSO shifted from 103.0 eV to 102.5 eV in PVA/CSO (Fig. 1n)^30^. Furthermore, the –OH stretching vibration peak of PVA molecules in the ATR–FTIR spectrum shifted from 3273 cm⁻¹ to 3296 cm⁻¹ in PVA/CSO (Supplementary Fig. 6)^31^. These spectroscopic shifts consistently indicate the formation of O–H⋯O hydrogen bonds between the –OH groups of PVA and the silicate groups of CSO, which is crucial for stabilizing the PVA/CSO ionic-molecular chain structure. The above results indicate that, under the guidance of PVA molecular chains, CSO undergoes 1D inorganic polymerization to form PVA/CSO composite ionic-molecular chains. These composite chains comprise crystalline and amorphous CaS units that are interconnected through hydrogen bonds formed between the –OH groups of the PVA molecular chains and the silicate units. Pronounced calcium-ion vacancies are present within the chains, whereas free Ca^2+^ ions are distributed in the interchain regions. The resulting PVA/CSO nanofibers assembled from ionic-molecular chains exhibit pronounced bending behavior, which provides the structural foundation for their macroscopic elastic deformation.

### Hierarchically integrated structure of PVA/CSO

**Fig. 2:**
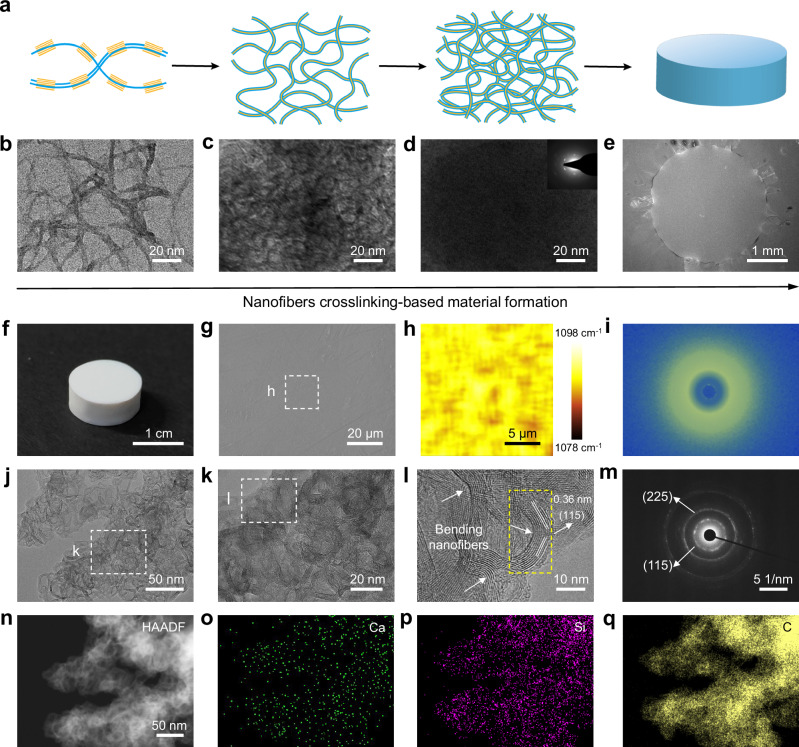
Hierarchical integrated structure of PVA/CSO. **a** Schematic illustration of the hierarchical assembly of the PVA/CSO bulk. **b**–**d** TEM images showing the PVA/CSO precursor slurry gradually crosslinking to form a continuous network during water evaporation (inset: corresponding SAED pattern). **e** SEM image of the PVA/CSO sheet obtained by natural drying of the precursor slurry. **f** Optical photograph of the PVA/CSO bulk. **g** Surface SEM image of the PVA/CSO bulk. **h** 2D Raman mapping of the Si–O characteristic peaks on the surface of the PVA/CSO bulk. **i** 2D SAXS pattern of the PVA/CSO bulk. **j**–**l** HRTEM images of an ultrathin section of the PVA/CSO bulk. **m** SAED pattern corresponding to (**j**). **n**–**q** HAADF-STEM micrograph of an ultrathin section of the PVA/CSO bulk and the corresponding elemental mapping of Ca, Si, and C.

The PVA/CSO nanofibers, formed by the assembly of PVA/CSO ionic-molecular chains, were subsequently employed to construct macroscopic bulk materials (Fig. 2a). Specifically, as moisture evaporated, the flexible PVA/CSO nanofibers in the homogeneous PVA/CSO precursor slurry (Supplementary Fig. 7) underwent crosslinking, forming a network structure analogous to molecular chain crosslinking in polymer elastomers (Fig. 2b, c). Especially, some PVA/CSO nanofibers tended to form bundle-like structures (Fig. 2b, Supplementary Fig. 8a, b), potentially due to the abundant hydroxyl groups in the PVA molecular chains on the nanofiber surfaces, which promote crosslinking and hydrogen bonding between adjacent nanofibers, driving the nanofibers to arrange closely and reduce the system’s surface energy. Elemental mapping analysis further confirmed the uniform distribution of Ca, Si, O, and C elements within the nanofiber bundle network (Supplementary Fig. 8c–f). As water continued to evaporate, the PVA/CSO slurry gradually transitioned into a gel state, with the internal nanofiber crosslinking network becoming denser (Fig. 2d). SAED pattern revealed distinct crystallization of CaS (inset in Fig. 2d). After the complete dehydration of the PVA/CSO gel, a continuous sheet with a smooth and flat surface was formed (Fig. 2e). Furthermore, by placing the PVA/CSO gel in a cylindrical mold and allowing it to dry naturally, a macroscopically uniform PVA/CSO bulk was obtained (Fig. 2f). SEM images revealed that the resulting PVA/CSO bulk exhibited a uniform and continuous dense structure across scales ranging from microns to millimeters (Fig. 2e, g and Supplementary Fig. 9a–c), while its cross-section showed flexible nanofibers assembled into ribbon-like morphologies (Supplementary Fig. 9d–f).

To further investigate the structural homogeneity of the PVA/CSO bulk, point-by-point micro-Raman mapping was conducted over a square region of its surface. The results revealed that the characteristic Si–O peak of the CaS phase (located at 1086.4 cm⁻¹) was uniformly distributed across the region, indicating that both the CaS phase and the hydrogen bonds between PVA molecular chains and the CaS phase were uniformly present on the surface of the PVA/CSO bulk (Fig. 2h, Supplementary Fig. 10a, b). 2D SAXS image exhibited a characteristic circular pattern (Fig. 2i), further confirming that the PVA/CSO bulk is isotropic, consistent with the molecular chain network characteristics of elastomers. High-resolution transmission electron microscopy (HRTEM) images of ultrathin sections of the PVA/CSO bulk revealed that the internal nanofibers exhibited a highly coiled morphology similar to that of polymer chains, intertwining to form a 3D crosslinked network (Fig. 2j–l), distinct from the rigid characteristics of conventional inorganic nanofibers. Lattice analysis of the curved nanofibers revealed a lattice spacing of 0.36 nm, corresponding to the (115) crystal plane of CaS. Furthermore, these nanofibers were not densely packed but retained nanoscale pores (Fig. 2j), a structural feature that will contribute to the apparent flexibility and elastic deformation capability of the PVA/CSO bulk at the macroscopic scale. SAED pattern revealed isotropic crystal planes (225) and (115) originating from CaS nanocrystals (Fig. 2m), further confirming the uniform distribution of the CaS phase within the coiled nanofibers. HAADF-STEM images and corresponding elemental mapping results clearly demonstrated the network structure formed by the crosslinking of PVA/CSO nanofibers, as well as the uniform dispersion of Ca and Si elements derived from the CaS phase and C elements derived from PVA chains within the network (Fig. 2n–q), confirming the structural homogeneity of the organic–inorganic crosslinked network in the PVA/CSO bulk at the nanoscale.

For comparison, CSO was replaced by CaS nanoparticles (CSNP, 100–200 nm, Supplementary Fig. 11) to prepare a PVA/CSNP bulk (Supplementary Fig. 12a). The resulting PVA/CSNP bulk exhibited an organic–inorganic composition ratio consistent with that of the PVA/CSO bulk (Supplementary Fig. 13). SEM images revealed that the resulting PVA/CSNP bulk exhibited significant agglomeration of CSNP on both its surface and interior (Supplementary Fig. 12b–f). In addition, distinct pores were formed within the material. This heterogeneous structure was attributed to the inability of CSNP to undergo 1D inorganic ionic polymerization, unlike CSO, making it difficult to form strong hydrogen bonds with PVA molecular chains and thus prone to phase separation. 1D and 2D Raman spectra further revealed significant fluctuations in the position of the Si–O peak on the surface of the PVA/CSNP bulk (Supplementary Fig. 10c, d), consistent with the phase-separated structure observed in the SEM images. This demonstrates that traditional nanocomposite strategies struggle to achieve an elastomer-like molecular chain crosslinked network, underscoring the unique role of CSO in constructing a mineral nanofiber crosslinked network that cannot be replicated by traditional nanomaterials. The results above demonstrate that the PVA/CSO bulk features an organic–inorganic hierarchically integrated crosslinked network structure. Under the induction of PVA molecular chains, CSO bypasses 3D inorganic ionic polymerization and instead undergoes 1D polymerization to form PVA/CSO composite ionic-molecular chains through hydrogen bonding with PVA molecular chains. These ionic-molecular chains, which have a calcium-deficient structure, further assemble into flexible, highly coiled nanofibers and bundles, ultimately forming the PVA/CSO bulk through entanglement and crosslinking in 3D space. This unique hierarchically integrated structure lays the structural foundation for its excellent plasticity and elastic deformation behavior at the macroscopic scale.

### Mechanical performance of PVA/CSO

**Fig. 3:**
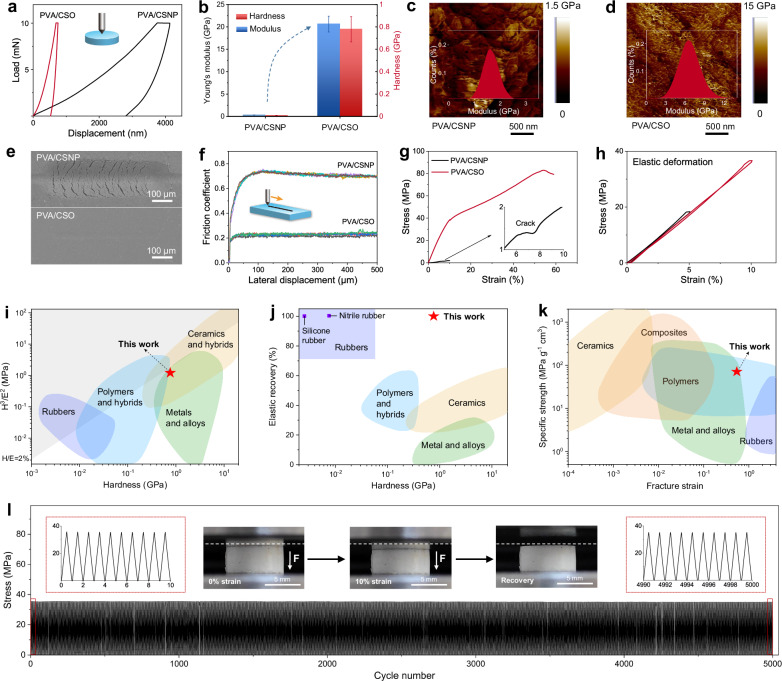
Mechanical performance of the PVA/CSO bulk. **a** Representative nanoindentation load–displacement curves of the PVA/CSNP and PVA/CSO bulks. **b** Surface hardness and Young’s modulus of the PVA/CSNP and PVA/CSO bulks. The data points shown are the mean of 3 independent measurements, and the error bars represent the standard deviation (SD) calculated from these repeated measurements. **c** DMT modulus mapping of the PVA/CSNP bulk. **d** DMT modulus mapping of the PVA/CSO bulk. The insets in (**c**) and (**d**) display the corresponding statistical distributions of the DMT modulus. **e** SEM micrographs showing scratches on the PVA/CSNP and PVA/CSO bulks after microscratch testing. **f** Friction coefficient versus lateral displacement curves for the PVA/CSNP and PVA/CSO bulks. **g** Representative compressive stress–strain curves of the PVA/CSNP and PVA/CSO bulks. The inset shows an enlarged view of the stress–strain curve of the PVA/CSNP bulk. **h** Stress–strain curves of the PVA/CSO bulk under cyclic compression within the elastic range, with a maximum compressive strain of 10%. **i** Comparison of H³/E² and H for the PVA/CSO bulk and other typical engineering materials, such as ceramics, ceramic-based composites, metals, alloys, rubbers, polymers, and polymer-based hybrids. The gray area represents the region where H/E ≥ 2%. Here, E denotes Young’s modulus, and H denotes hardness. **j** Comparison of elastic recovery rate and H between the PVA/CSO bulk and typical ceramics, metals, and alloys, rubbers, polymers, and polymer-based hybrids. **k** Comparison of specific strength and fracture strain between the PVA/CSO bulk and ceramics, metals, alloys, rubbers, polymers, and organic–inorganic composites. The mechanical data of these reference materials are derived from the previous report^40^. **l** Stress–cycle number curve of the PVA/CSO bulk in the cyclic compression test. The side insets display the stress–cycle number curves for the PVA/CSO bulk during the first and last 10 cycles of the 5000-cycle test. The central insets present optical photographs of the PVA/CSO bulk during a single compression cycle. The compression rate was 2 mm min⁻¹, with a maximum compressive strain of 10% and a total of 5000 cycles.

The internal structure of a material usually plays a decisive role in its mechanical properties^32,33^. Therefore, the PVA/CSO bulk can exhibit distinctive mechanical behaviors due to its unique crosslinked network structure of flexible nanofibers. First, the micromechanical properties of the PVA/CSO bulk were evaluated through nanoindentation testing, using the PVA/CSNP bulk as a control. Representative load–displacement curves are presented in Fig. 3a. The results indicate that, under the same applied load, the indentation depth of the PVA/CSO bulk is substantially smaller than that of the PVA/CSNP bulk. Further calculations show that the hardness (H) and Young’s modulus (E) of the PVA/CSO bulk are 0.78 ± 0.11 GPa and 20.63 ± 1.77 GPa, respectively (Fig. 3b), which are significantly higher than those of the PVA/CSNP bulk. These results suggest that, compared with the heterogeneous structures arising from phase separation in traditional nanocomposites, the uniform network formed through ionic-molecular assembly imparts higher hardness and Young’s modulus to the material. Especially, the PVA/CSO bulk exhibited a small hysteresis loop during the loading–unloading process, indicating that it not only undergoes plastic deformation under stress but also exhibits elastic recovery similar to elastomers. Subsequently, the indentation region on the surface of the PVA/CSO bulk was examined using SEM. The result shows that only slight traces remain at the indentation site (Supplementary Fig. 14), with no brittle fracture phenomena commonly observed in traditional mineral materials, further confirming the excellent toughness and elastic deformation capability of PVA/CSO bulk. Additionally, nanomechanical characterization of 2.5 μm × 2.5 μm surface regions of the PVA/CSNP and PVA/CSO bulks was conducted using the PeakForce Quantitative Nanomechanical Mapping (PF-QNM) mode of atomic force microscopy (AFM). The results indicate that the PVA/CSO bulk displays a more uniform surface morphology. Statistical analysis shows that its Derjaguin–Müller–Toporov (DMT) modulus reaches 6.6 GPa, which is significantly higher than the 1.6 GPa measured for the PVA/CSNP bulk (Fig. 3c, d). This finding is consistent with the nanoindentation results, collectively demonstrating that the PVA/CSO bulk possesses higher surface stiffness.

Subsequently, micro-scratch tests were performed on the PVA/CSNP and PVA/CSO bulks. The results indicate that, under the same applied load, the contact depth and residual depth of the PVA/CSO bulk are significantly smaller than those of the PVA/CSNP bulk (Supplementary Fig. 15). SEM images further reveal that the surface of the PVA/CSO bulk undergoes only slight plastic deformation with no evident residual scratches, whereas the PVA/CSNP bulk displays pronounced brittle cracking behavior (Fig. 3e). These findings demonstrate that, compared with the PVA/CSNP bulk, the PVA/CSO bulk possesses higher surface hardness, modulus, and toughness, as well as superior elastic recovery capability. The calculated friction coefficient reveals that the average friction coefficient of the PVA/CSO bulk is 0.23, substantially lower than the 0.71 measured for the PVA/CSNP bulk (Fig. 3f), reflecting its excellent anti-wear performance. This performance is primarily attributed to its internally crosslinked and mechanically robust nanofiber network, suggesting that the PVA/CSO bulk has broad application potential in the field of high-performance anti-wear materials.

Given the excellent toughness and elastic deformation capability exhibited by the PVA/CSO bulk at the microscale owing to its crosslinked nanofiber network, it suggests that the material is expected to exhibit distinctive toughness at the macroscale. To verify this, macroscopic compression tests were conducted on the PVA/CSNP and PVA/CSO bulks. Representative compressive stress–strain curves are presented in Fig. 3g. The stress–strain curve of the PVA/CSNP bulk exhibits significant fluctuations once the compressive strain exceeds 6%, indicating brittle cracking within the material; when the strain reaches 10%, the structure collapses completely (Supplementary Movie 1). In contrast, the PVA/CSO bulk shows a linear stress–strain relationship at strains below 10%, indicating elastic deformation. As the strain increases from 10% to 50%, the curve remains smooth and continuous, signifying substantial plastic deformation, while further increased strain results in a pronounced stress drop, marking the onset of fracture. Its peak compressive strength reaches 82.0 MPa, far exceeding that of the PVA/CSNP bulk (1.4 MPa). Meanwhile, the elastic modulus of the PVA/CSO bulk is 380 ± 14 MPa, which is also significantly higher than that of the PVA/CSNP bulk (22 ± 2 MPa). To further evaluate its elastic behavior, we conducted single-cycle compression tests on the PVA/CSO bulk with maximum strains set at 5% and 10%, respectively. The results show that the loading and unloading curves essentially overlap, with no significant hysteresis. Dynamic video recordings further demonstrate that the PVA/CSO bulk undergoes typical reversible elastomeric deformation during compression, without the brittle fragmentation observed in brittle minerals (Supplementary Movie 2), indicating nearly complete elastic recovery within a 10% strain range.

A comparison shows that the H³/E² value of the PVA/CSO bulk is 1.12 MPa. This parameter reflects the material’s resistance to plastic deformation^34,35^, and the obtained value is comparable to those of ceramics and significantly higher than those of most polymers and rubbers. Its hardness is comparable to that of certain ceramics and even exceeds that of some metal systems (Fig. 3i), reflecting an excellent combination of high hardness and wear resistance. Additionally, the PVA/CSO bulk achieves 100% elastic recovery at a 10% compressive strain, comparable to rubber and significantly higher than ceramics, metals, alloys, and most polymer nanocomposites (Fig. 3j). Its specific strength reaches 74.53 ± 3.56 MPa g⁻¹ cm³, comparable to common ceramics and polymers and substantially higher than rubber (Fig. 3k). Therefore, the PVA/CSO bulk combines the high strength of minerals with the high resilience and fracture strain of elastomers, thus being termed an elastic mineral.

To further evaluate its durability, we conducted 5000 cyclic compression tests on the PVA/CSO bulk at a maximum compressive strain of 10%. The results show that as the number of cycles increased from 0 to 5000, the peak compressive stress remained stable without any detectable decay, indicating that the PVA/CSO bulk retains its structural integrity under repeated loading (Fig. 3l). This cyclic stability further confirms the excellent elastic recovery and anti-fatigue performance of PVA/CSO as an elastic mineral. Overall, these findings demonstrate that, owing to its unique crosslinked nanofiber network, the PVA/CSO bulk integrates multiple advantageous properties such as high hardness, high modulus, high toughness, superior elastic recovery, and high fatigue resistance, thereby broadening its application potential in high-performance elastic materials.

### Moldability and functionalization of PVA/CSO

**Fig. 4:**
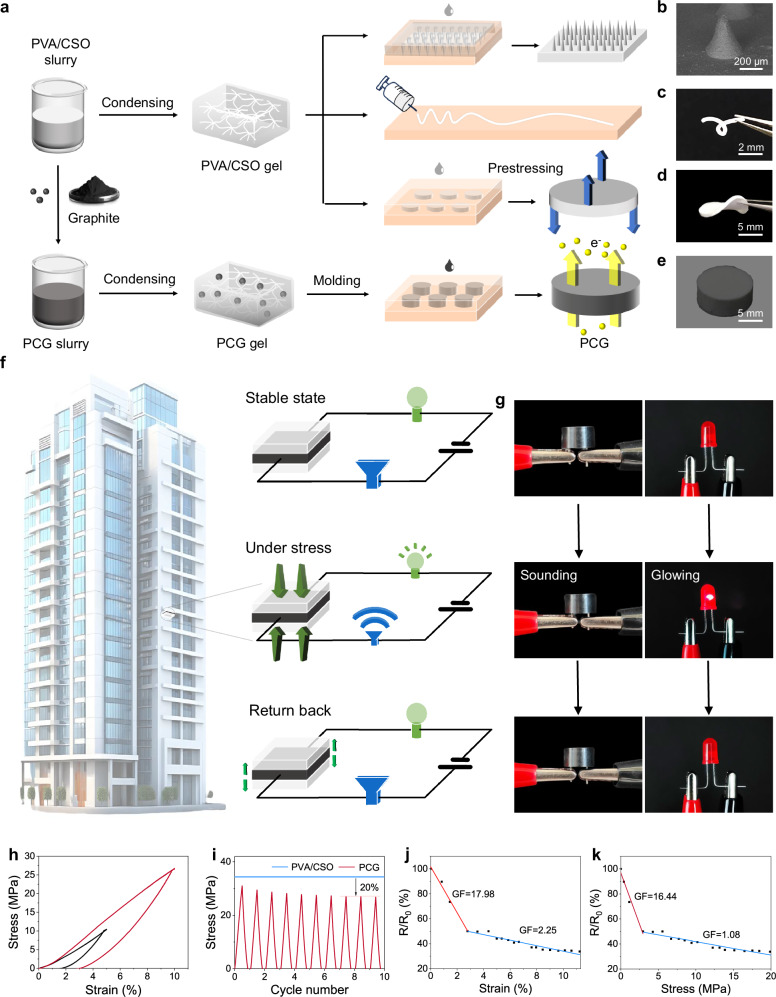
Moldability and functionalization of PVA/CSO elastic minerals. **a** Schematic illustration of the moldable process of the PVA/CSO elastic minerals. The PVA/CSO precursor slurry can be processed through dehydration-induced gelation, followed by injection or casting molding, to form bulk phases with complex 3D architectures across micrometer-, millimeter-, and centimeter-scales. By incorporating conductive graphite into the PVA/CSO precursor slurry, a smart PCG elastic mineral can be produced for use as a strain sensor. **b**–**d** Molded PVA/CSO bulks exhibiting various dimensions and 3D morphologies. **e** Optical photograph of the PCG bulk. **f** Schematic illustration of the working mechanism of the PCG sensor for stress (strain) response and warning. **g** Optical photographs showing the PCG sensor conducting electricity under stress to activate a buzzer and warning light, with the circuit disconnecting upon stress release. **h** Compressive stress–strain curves of the PCG sensor during a single loading–unloading cycle under 5% and 10% compressive strains. **i** Stress–cycle number curve of the PCG sensor over 10 cycles at a compressive strain of 10%. **j** Variation in the relative resistance of the PCG sensor as a function of strain under different compressive loads. **k** Variation in the relative resistance of the PCG sensor as a function of stress under different compressive loads.

Unlike traditional mineral materials (such as ceramics and cement), which rely on high-temperature sintering for processing^36,37^, the molding process of PVA/CSO elastic minerals is more environmentally friendly and low-carbon, possessing multi-scale plastic processing and functionalization capabilities. As shown in Fig. 4a, after evaporative dehydration, the uniform PVA/CSO precursor slurry forms a gel. This gel consists of an internal crosslinked nanofiber network, endowing it with excellent moldability, facilitating shaping, and enabling the fabrication of continuously structured integral components. In terms of micro-scale precision applications, micron-level PVA/CSO microneedles can be prepared using microneedle molding molds (Fig. 4b), demonstrating their potential in the field of fine structures. At the millimeter scale, curled fiber-like structure can be obtained through injection extrusion processes (Fig. 4c), demonstrating the feasibility of constructing complex components using 3D printing technology. Furthermore, by applying pre-stress in different directions to the initially formed PVA/CSO gel sheets (with a water content of approximately 25%) during the drying process, complex centimeter-scale curled sheet structure can be produced (Fig. 4d), fully illustrating their exceptional plasticity and processing adaptability. This multiscale plastic molding capability arises from the unique properties of the nanofiber crosslinked network, enabling PVA/CSO elastic minerals to adopt processing pathways similar to those of polymers, thus offering an effective method for constructing complex elastic mineral structural components.

Additionally, by introducing functional nanomaterials (e.g., conductive graphite) into the PVA/CSO precursor slurry, a PCG gel is produced, which, after mold forming, yields a black conductive elastic mineral (Fig. 4e). SEM images demonstrate a continuous and dense surface of the resulting PCG elastic mineral, comparable to that of the PVA/CSO bulk. The cross-sectional view clearly shows graphite nanoparticles uniformly distributed within the PVA/CSO network, providing the structural foundation for the formation of conductive pathways under compressive stress (Supplementary Fig. 16). To explore the practical applications of PCG, it was integrated as a strain sensor module into a circuit, along with an alarm indicator light and buzzer module, to construct a smart warning system (Fig. 4f). This system can be installed at critical stress-bearing locations in large-scale construction projects (e.g., high-rise buildings and bridges). In the absence of external force, the graphite particles within the PCG module do not form an effective conductive pathway, and the circuit remains open. When subjected to external pressure, the 3D network inside the PCG undergoes elastic-plastic deformation, causing the graphite particles to connect and form electron conduction paths, leading to a significant decrease in resistance, circuit conduction, and the triggering of audible and visual alarms. Upon unloading the pressure, the 3D network recovers reversibly, and the circuit reopens (Fig. 4g, Supplementary Movie 3).

Cyclic compression tests were further conducted on the PCG elastic mineral, with maximum strains of 5% and 10%. The results revealed that, compared to the fully elastic deformation of the PVA/CSO bulk (Fig. 3h), the introduction of graphite particles caused hysteresis loops in the stress–strain curve of PCG, indicating that its elastic network was somewhat affected, with an elastic recovery rate of approximately 68.4% (Fig. 4h). Under 10% strain over 10 consecutive compression cycles, PCG maintained good elasticity, with a stable peak stress of 26.8 MPa. Although this is approximately 20% lower than that of PVA/CSO (Fig. 4i), the overall strength and elastic strain range remained high, making it suitable for structural health monitoring in civil engineering under challenging conditions such as bridge overloads and earthquake warnings. More importantly, PCG integrates the high strength and wear resistance of mineral materials, the reversible deformation capability of polymer elastomers, and electrical sensing functionality into a single material system, thereby achieving multifunctional integration of load-bearing and sensing. This characteristic enables long-term, stable strain perception in harsh service environments characterized by high wear and heavy loads, thereby overcoming the limitations of conventional purely polymer-based flexible sensors in terms of mechanical durability and load-bearing capacity. Leveraging this advantage, PCG demonstrates broad application prospects in smart structures and intelligent equipment. It can be employed not only for health monitoring and predictive maintenance of critical large-scale equipment, such as wind turbine blades, turbines, pipeline systems, and aerospace structural components, but also integrated into robotic grippers to provide high-precision tactile perception, thereby significantly enhancing operational intelligence and safety.

For compressible sensors, a wide sensing range and high sensitivity are critical. The sensing performance of PCG was assessed by plotting the relationship curves between the relative change in resistance and compressive stress/strain (Fig. 4j, k). The slope of the curve, referred to as the gauge factor (GF), was used to quantify sensitivity^38^. The results indicate that PCG exhibits a two-stage resistance response: in the low strain range (0–2.8%) and the low stress range (0–3.0 MPa), the GF values reach 17.98 and 16.44, respectively, with sensitivity significantly higher than in the high strain/stress regions. This phenomenon is attributed to the more effective establishment of new conductive pathways through internal network reconstruction under low strain/stress conditions, resulting in significant changes in resistance and consequently higher sensitivity. In contrast, under high strain/stress conditions, circuit conduction stabilizes, resistance changes slow down, and the GF values decrease correspondingly. It is noteworthy that the graphite content exerts a decisive influence on the sensing performance of PCG. In this study, the graphite content in PCG was optimized to 10.0 wt%. When the graphite content is too low, the particles fail to form a continuous conductive network, rendering PCG electrically insulating or characterized by excessively high resistance, thereby preventing effective sensing. Conversely, when the graphite content is excessive, the conductive network becomes overly dense, enabling PCG to maintain high conductivity even in the unstrained state, which results in a limited resistance variation range and reduced sensitivity. More critically, excess graphite tends to aggregate, disrupting the integrity of the PVA/CSO ionic-molecular crosslinked network, generating stress concentration sites, inducing microcracks, and reducing the elastic recovery capability of the material, thereby increasing brittleness. Once irreversible deformation occurs, the stability and accuracy of the sensor are significantly compromised. Therefore, an optimal graphite content of 10.0 wt% enables PCG to simultaneously achieve excellent mechanical strength, high sensitivity, a wide sensing range, and excellent cyclic stability. With its excellent elasticity and high sensitivity, the PCG conductive elastic mineral can hence serve as a strain sensor, enabling real-time warnings in dynamic load environments and demonstrating broad application prospects in the field of structural engineering.

## Discussion

In summary, we propose a 1D inorganic ionic polymerization tactic for the construction of elastic minerals. Guided by PVA molecular chains, CSO undergoes 1D polymerization, resulting in highly flexible PVA/CSO composite ionic-molecular chains. These chains are hierarchically assembled into nanofibers and bundles, which ultimately crosslink to form a 3D network. This unique structure imparts to the PVA/CSO elastic mineral the exceptional mechanical properties of inorganic ionic minerals, while also exhibiting over 50% compressive plastic deformation. After 5000 cycles at 10% compressive strain, the PVA/CSO elastic mineral demonstrates complete structural recovery and maintains elastic stability. Furthermore, by incorporating conductive functional units into the PVA/CSO elastic mineral system, a smart PCG elastic mineral was developed, capable of functioning as a strain sensor for real-time early warning in dynamic load environments, such as earthquakes and bridge overloads, thereby demonstrating broad application potential in structural engineering. The proposed 1D inorganic ionic polymerization tactic not only deepens the understanding of elastic mineral concepts and structural design but also breaks the boundaries between traditional inorganic minerals and polymer elastomers, paving the way for the development of high-performance and multifunctional elastic minerals.

## Methods

### Materials

Calcium chloride dihydrate (CaCl_2_·2H_2_O) was purchased from Sigma-Aldrich. Triethylamine (TEA) was purchased from Sinopharm Chemical Reagent Co., China. Polyvinyl alcohol (PVA, [−CH_2_CHOH − ]_n_, alcoholysis degree: 98.0 ~ 99.0 mol%, viscosity: 54.0 ~ 66.0 mPa·s), dimethyl sulfoxide (DMSO, 99.5%), α-Cyano-4-hydroxycinnamic acid (HCCA), and calcium silicate nanoparticles (CSNP) were purchased from Aladdin’s Reagent Co., China. Graphite and tetraethyl orthosilicate (TEOS) were purchased from Macklin Biochemical Technology Co., Ltd., China. Ethanol absolute (≥ 99.7%) was purchased from Shanghai Titan Scientific Co., China. Standard hydrochloric acid solution (HCl, 1 mol L^−1^) was purchased from Tianjin Jiangtian Chemical Technology Co., China. All chemicals were used as received without further purification.

### Synthesis of calcium silicate oligomers (CSO)

CSO was synthesized as follows. First, 13.44 mL of TEOS was added to a solution consisting of 35.1 mL of ethanol, 10.2 mL of deionized water, and 0.6 mL of HCl (1 mol L⁻¹). The hydrolysis was conducted for 1 h to yield the TEOS hydrolysate. Subsequently, 8.82 g of CaCl₂·2H₂O was dissolved in 3 L of ethanol with vigorous stirring to obtain a clear calcium salt-ethanol solution. Then, 166.8 mL of TEA was added to this solution, followed by vigorous stirring at 25 °C for 30 min. Subsequently, the TEOS hydrolysate was added dropwise to the reaction system, and stirring was maintained at 25 °C for 12 h. Following the reaction, the resulting slurry was centrifuged at 3250 × *g* for 5 min to collect the CSO precipitate. The precipitate was washed three times with ethanol to remove residual TEA. Finally, the CSO precipitate was redispersed in ethanol to achieve a homogeneous dispersion at a concentration of 20 mg mL⁻¹ for future use.

### Preparation of PVA/CSO elastic mineral

First, 100 mL of a CSO dispersion (20 mg mL⁻¹) was centrifuged at 3250 × *g* for 5 min to collect the precipitate. This centrifugation speed effectively precipitates CSO of approximately 1.5 nm while retaining smaller soluble species in the supernatant, thereby avoiding yield loss from excessively low speeds and redispersion difficulties caused by overly compacted precipitates at excessively high speeds. Subsequently, the precipitate was mixed with 20 mL of a PVA aqueous solution (3.0 wt%) and stirred vigorously for 3 h to obtain a homogeneous PVA/CSO slurry. The slurry was then transferred to a culture dish and dried at 25 °C until it lost fluidity and transitioned into a gel state, thus yielding the PVA/CSO precursor gel with a water content of about 25%. Finally, the gel was injected into specific molds and naturally dried at 25 °C to obtain the PVA/CSO elastic mineral.

### Preparation of PVA/CSNP bulk

The PVA/CSNP bulk was prepared through the following procedure. First, 2 g of CSNP was added to 8 g of water to form a 20 wt% CSNP dispersion. Subsequently, 20 mL of a PVA aqueous solution (3.0 wt%) was uniformly mixed with this dispersion to form a PVA/CSNP composite slurry. The resulting slurry was then transferred to a culture dish and dried at 25 °C until it reached a viscous state. This viscous slurry was subsequently centrifuged at 13,000 × *g* for 10 min, and the supernatant, containing unbound PVA molecules, was discarded to obtain a doughy PVA/CSNP precursor gel. Finally, this gel was injected into specific molds and dried at 25 °C to yield the final PVA/CSNP bulk.

### Preparation of PVA/CSO/graphite (PCG) smart elastic mineral

First, 100 mL of CSO dispersion (20 mg mL⁻¹) was centrifuged at 3250 × *g* for 5 min, after which the supernatant was discarded and the precipitate was collected. Subsequently, 20 mL of a PVA aqueous solution (3.0 wt%) was added to the precipitate and vigorously stirred for 3 h to form a homogeneous PVA/CSO slurry. Next, 0.29 g of graphite was incorporated into the slurry, and stirring was continued for an additional 3 h to obtain a homogeneous black slurry. This slurry was subsequently transferred to a culture dish and dried at 25 °C until it ceased flowing and gelled, thereby yielding the PCG precursor gel with a water content of about 25%. Finally, the gel was injected into specific molds and dried at 25 °C to obtain the final smart PCG elastic mineral.

### X-ray diffraction (XRD)

XRD patterns were acquired on an X’Pert PRO diffractometer (PANalytical, Netherlands) employing Cu Kα radiation (*λ* = 1.54 Å). The measurements were conducted at 40 kV and 40 mA, scanning the 2θ range from 10° to 60° in continuous mode with a scan rate of 5° min⁻¹.

### Mass Spectrometry (MS) Analysis

High-resolution mass spectra were acquired using a matrix-assisted laser desorption/ionization time-of-flight mass spectrometer (MALDI-TOF MS, Bruker, Germany), equipped with both MALDI (matrix-assisted laser desorption/ionization) and TOF (time-of-flight) technologies. HCCA was chosen as the matrix, and a dilute CSO dispersion was utilized for MS analysis. All data presented were processed with background subtraction.

### Thermogravimetric analysis (TGA)

The samples were analyzed using a thermogravimetric analyzer (TGA/DSC3 + , METTLER TOLEDO, Switzerland). The measurements were conducted under a constant air flow rate of 50 mL min⁻¹, with a heating rate of 10 °C min⁻¹ from 50 °C to 800 °C.

### Inductively coupled plasma optical emission spectrometry (ICP-OES)

After dissolution with HCl and volumetric dilution, the concentrations of Ca²⁺ and SiO_3_²⁻ in the PVA/CSO nanofibers were determined using an inductively coupled plasma optical emission spectrometer (ICP‑OES; model 5110, Agilent Technologies, USA). The molar ratios were then calculated accordingly.

### Dynamic light scattering (DLS)

The hydrodynamic diameter distribution of CSNP was measured using a dynamic light scattering photometer (Zetasizer Nano ZS90; Malvern Instruments, USA). A CSNP dispersion with a concentration of 0.5 mg mL⁻¹ was directly analyzed at 25 °C.

### X-ray photoelectron spectroscopy (XPS) analysis

XPS measurements were conducted using a K-Alpha X-ray Photoelectron Spectrometer (Thermo Scientific, ESCALAB-Xi, USA) to obtain high-resolution XPS spectra of Si 2p. A monochromatic aluminum X-ray source (Al Kα = 1486.6 eV) was used for excitation, with the operating voltage and current set to 12 kV and 6 mA, respectively. All binding energies were calibrated using the standard C 1 s peak at 284.6 eV as a reference point.

### Attenuated total reflectance-Fourier transform infrared spectroscopy (ATR–FTIR)

ATR–FTIR spectra were obtained using a Fourier Transform Infrared Spectrometer equipped with an attenuated total reflectance accessory (FTIR-iS50, FEI, USA). The spectra were recorded directly at 25 °C over a wavenumber range of 400–4000 cm⁻¹, with the final spectra obtained by accumulating 32 scans.

### Raman spectroscopy

Raman spectra were acquired using a laser confocal micro-Raman spectrometer (LabRAM HR Evolution; Horiba Scientific, France) equipped with a 532 nm laser excitation source. Raman spectra of ethanol solutions of TEA, TEA/CaCl₂, TEA/TEOS, TEA/TEOS hydrolysate, and CSO dispersion, as well as bulk samples of PVA/CSO, PVA/CSNP, and CSO, were recorded directly at 25 °C over a range of 100–2000 cm⁻¹.

### Nuclear Magnetic Resonance (NMR) Spectroscopy

Liquid NMR spectra were acquired using a 600 MHz NMR spectrometer (AVANCE III, Bruker, Germany). A 0.2 mL aliquot of a diluted CSO dispersion was introduced into an NMR tube containing DMSO-d⁶, equipped with a sealed capillary. The ²⁹Si spectrum was recorded immediately at 25 °C. TEOS solution and TEOS hydrolysate solution were analyzed in parallel as control groups using the same procedure.

### Small-angle X-ray scattering (SAXS)

SAXS measurements were conducted on a Xeuss 2.0 SAXS/WAXS system (Xenocs, France) operated at 50 kV and 0.60 mA. A Cu Kα radiation source with a wavelength of 1.54189 Å was used, and the sample-to-detector distance was set to 539 mm. Scattering signals were collected using a Pilatus 3 R 300 K detector with a data acquisition time of 300 s per sample. The obtained 2D scattering patterns were azimuthally integrated to yield 1D profiles representing the relationship between scattering vector (*q*) and scattering intensity. Raw data were processed using the BioXTAS RAW software package. To enhance peak resolution, the scattering data were analyzed by plotting *I*(*q*)∙*q*² versus *q*, and the interdomain spacing (*L*) was calculated using Bragg’s law^39^:

1 $$L=2{{{\rm{\pi }}}}/{q}_{\max }$$

where *q*_*max*_ denotes the scattering vector corresponding to the maximum peak intensity.

### Morphology and microstructure characterization

The morphology and microstructure of the samples were characterized using a field emission scanning electron microscope (FESEM, SU8600, Hitachi, Japan) at an acceleration voltage of 5 kV, a transmission electron microscope (TEM, JEM-F200, JEOL, Japan), and a spherical aberration-corrected transmission electron microscope (Cs-TEM, JEM-ARM300F2 WGP, JEOL, Japan). Energy dispersive spectroscopy (EDS) analysis was conducted using an analysis station (JED-2300T, JEOL, Japan).

### Nanoindentation testing

Nanoindentation measurements were performed using a nanoindenter (G200, Agilent Technologies, CA, USA) equipped with a Berkovich tip (tip radius ≈ 20 nm). All tests were conducted under controlled conditions of 25 °C and 40% relative humidity, with the tip calibrated using fused silica as a reference. The hardness and Young’s modulus of the samples were determined using the constant load measurement method. During the loading process, a constant load of 10 mN was applied at a controlled strain rate of 0.2 nm s⁻¹. The applied load and penetration depth were continuously monitored by the computer system throughout the indentation process. Ten indentation points were tested for each sample. Data were acquired and processed using TestWorks 4 software (MTS Systems Corporation, Eden Prairie, MN, USA), and the hardness and Young’s modulus values were derived from the average of the force-displacement curves.

### Microscratch testing

Microscratch tests were conducted using a microscratch tester (WS-2005, Anton Paar NHT + MCT, Austria). A Rockwell diamond indenter with a tip radius of 50 μm was used for all tests. The samples, prepared to ensure consistent surface roughness through polishing with high-precision sandpaper, were tested under a constant normal load of 0.5 N at a scratching speed of 1 mm min⁻¹ over a scratch length of 1 mm. Four parallel scratches were generated in each test region, with a minimum separation of 500 μm between adjacent scratches.

### Atomic force microscopy (AFM) testing

AFM images of the residual indentations for all samples were acquired using a multimode atomic force microscope (Dimension Icon, Bruker, Germany) in tapping mode under ambient conditions (25 °C, 40% relative humidity). All images and modulus distributions were analyzed and exported using the image analysis software (NanoScope Analysis, Version 3.00).

### Macroscopic compression testing

Compression experiments were conducted using an electronic universal testing machine (UTM2503, Shenzhen Suns Technology Stock Co., Ltd., China). Prior to testing, the specimens (8 mm in diameter, 5 mm in height) were dried at 37 °C for 24 h and subsequently conditioned at 25 °C and 40% relative humidity for 24 h. Quasi-static compression tests were performed at a rate of 2 mm min⁻¹, with the mechanical properties of each sample determined from the average of three parallel specimens. Cyclic compression tests were carried out at the identical rate of 2 mm min⁻¹ under a maximum compressive strain of 10% for 5000 cycles, during which the stress–cycle number curves were recorded throughout the compressive cycling process. The elastic recovery rate was calculated based on the cyclic loading–unloading curves. For each loading–unloading cycle, the maximum applied strain (*ε*_*max*_, which is 10% in this case) and the residual strain upon unloading when the stress returns to zero (*ε*_*res*_) were recorded. The elastic recovery rate (*R*_*ε*_) was calculated using the following equation:

2 $${R}_{\varepsilon }=({\varepsilon }_{\max }-{\varepsilon }_{{res}})/{\varepsilon }_{\max }\times 100\%$$

### Resistance-strain response

Electrical measurements were performed using a MEIRUIKE RK2516 DC low-resistance tester coupled with an electronic universal testing machine (UTM2503, Shenzhen Suns Technology Stock Co., Ltd., China). During testing, the PCG sample was connected to the measurement circuit, and compression tests were conducted at a rate of 2 mm min⁻¹. Within the elastic deformation range (compressive strain ≤ 10%), the PCG sample was compressed until circuit conduction occurred, establishing the relationship between strain/stress and resistance to validate the application performance of PCG under dynamic load conditions. The corresponding relative resistance change (Δ*R*/*R*₀) and sensitivity (GF) under varying strain/stress conditions were calculated according to Eqs. (3) and (4), respectively^38^.

3 $$R/{{{{\rm{R}}}}}_{0}=(R-{{{{\rm{R}}}}}_{0})/{{{{\rm{R}}}}}_{0}$$

4 $${GF}=(\triangle R/{{{{\rm{R}}}}}_{0})/\triangle P$$

where R_0_ is the resistance without strain applied, R is the resistance with strain applied, and ΔP is the stress (strain) change.

## Supplementary information

Supplementary Information

Description of Additional Supplementary Files

Supplementary Movie 1

Supplementary Movie 2

Supplementary Movie 3

Peer Review file

## Source data

Source Data

## Data Availability

All data are available in the main text or the Supplementary Information. Source data are provided with this paper.
