# Supplementary material for: One-dimensional inorganic ionic polymerization for elastic minerals
Source: Nat Commun. 2026 May 27;17:6903. doi: 10.1038/s41467-026-72767-0 (PMC13389460; doi:10.1038/s41467-026-72767-0)
Supplement: Supplementary file 1 — Supplementary Information [file 41467_2026_72767_MOESM1_ESM.pdf]

Supplementary Information

One-dimensional inorganic ionic polymerization for elastic minerals

Yongjin Du[1#], Zeyu Gong[1#], Ruoyan He[1], Manfang Hu[1], Lina Zhou[1], Wenge Jiang[2*], Yadong Yu[1*], and Junbo Gong[1*]

[1] School of Chemical Engineering and Technology, Tianjin University, Tianjin, 300072, China

[2] Department of Chemistry, Tianjin University, Tianjin, 300072, China

#These authors contributed equally to this work.

*Corresponding Authors Emails: wenge.jiang@tju.edu.cn; yuyd92_@tju.edu.cn; junbo_gong@tju.edu.cn

This Supplementary Information file includes:

Supplementary Figure 1–16

Supplementary Figures

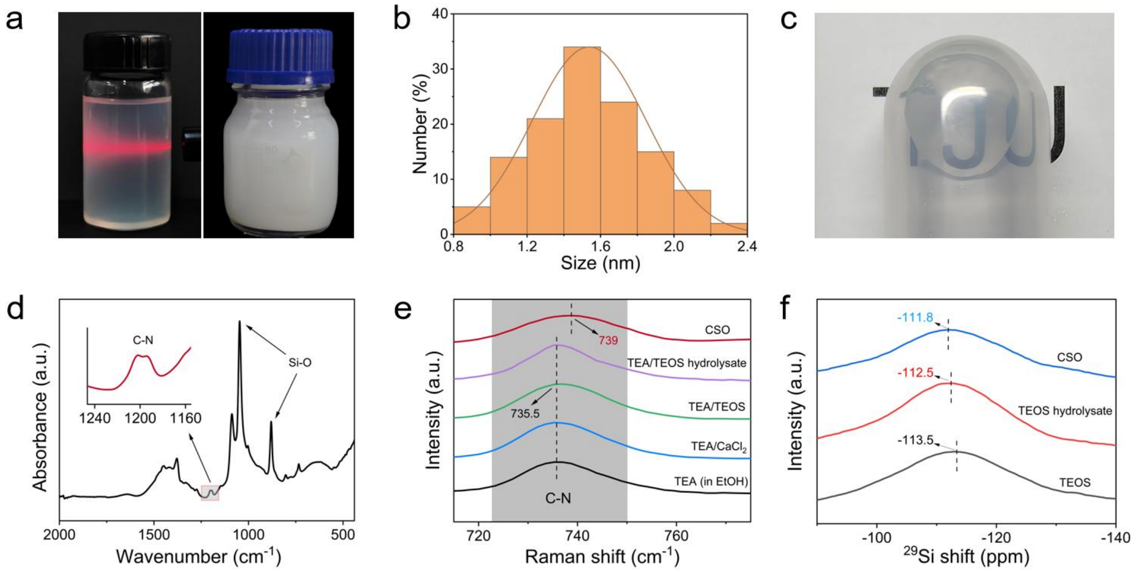

Supplementary Fig. 1. (a) Optical photograph of CSO dispersed in ethanol at the concentration of 0.1 mg mL^{-1} (left) and 20 mg mL^{-1} (right). (b) Size distribution of CSO, as statistically determined from the TEM image in Fig. 1b. (c) Optical photograph of the CSO gel obtained by centrifugation of the CSO ethanol dispersion at 8,000 rpm for 5 min. (d) ATR-FTIR spectra of the CSO gel. The inset shows a partially enlarged detail. (e) Liquid-phase Raman spectra of TEA, TEA/$CaCl_2$ mixture, TEA/TEOS mixture, TEA/TEOS hydrolysate, and CSO. (f) ^{29}Si liquid-state NMR spectra of TEOS, TEOS hydrolysate, and CSO.

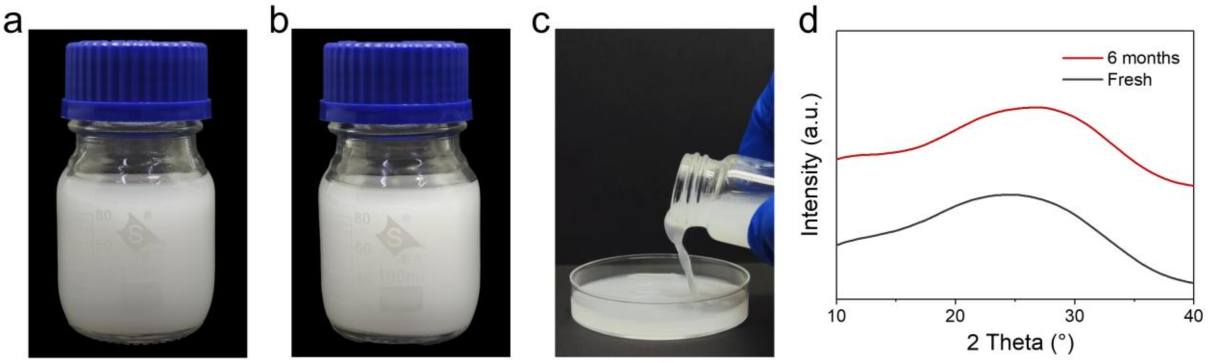

Supplementary Fig. 2. (a, b) Optical photographs of CSO dispersed in ethanol (20 mg mL^{-1}): (a) freshly prepared and (b) after approximately 6 months of storage. (c) Optical photograph of the CSO dispersion after approximately 6 months of storage, showing good fluidity. (d) XRD pattern of freshly prepared CSO and CSO after approximately 6 months of storage.

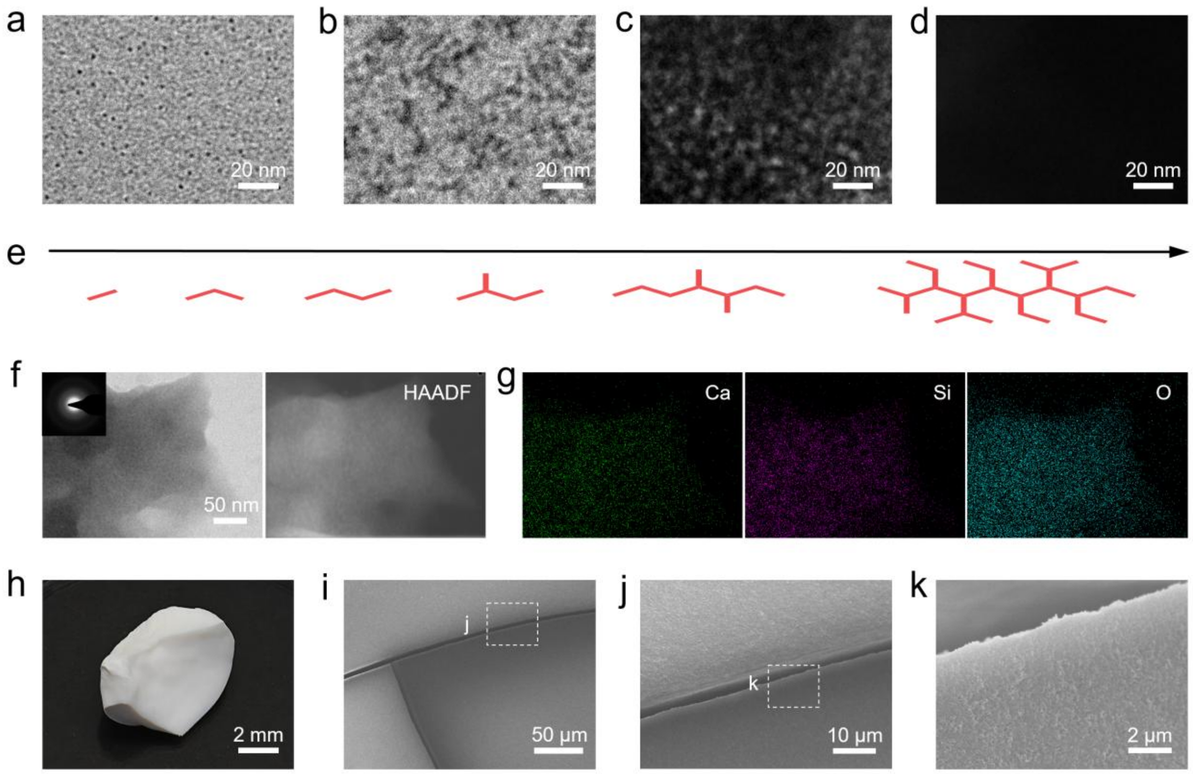

Supplementary Fig. 3. (a–d) TEM images showing the spontaneous crosslinking of CSO via inorganic ionic polymerization in an ethanol dispersion as TEA and the solvent evaporate. (e) Corresponding schematic illustration of the chain growth of CSO through inorganic ionic polymerization. (f) TEM and HAADF-STEM images of the continuous CaS network formed through inorganic ionic polymerization of CSO. Inset is the SAED pattern. (g) Corresponding element mapping of Ca, Si, and O of the continuous CaS network. (h) Optical photograph of the CaS bulk obtained through drying the CSO gel. (i–k) SEM images of the corresponding CaS bulk.

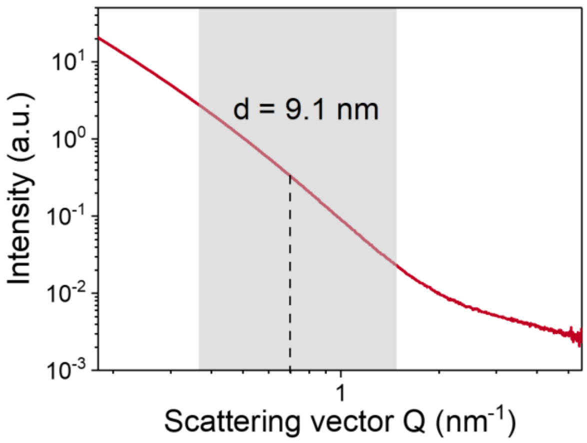

Supplementary Fig. 4. 1D SAXS pattern of the ionic-molecular chains in the PVA/CSO dispersion.

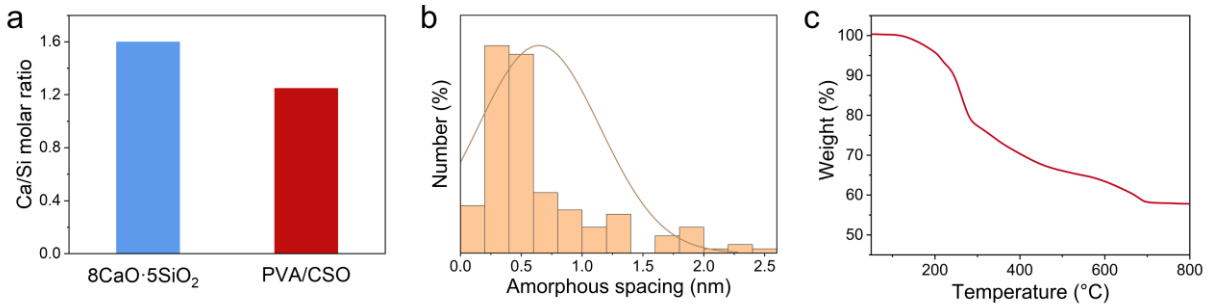

Supplementary Fig. 5. (a) Ca/Si molar ratio in the PVA/CSO nanofibers as determined by ICP-OES, compared with that in the standard crystalline compound $8CaO{\cdot}5SiO_2$. (b) Length distribution of amorphous regions containing vacancies in the PVA/CSO ionic−molecular chains, statistically analyzed from the TEM image in Fig. 1h. (c) TGA curve of the PVA/CSO nanofibers.

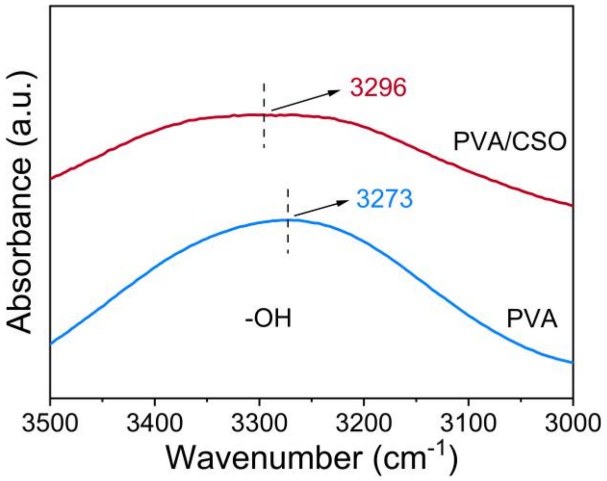

Supplementary Fig. 6. ATR-FTIR spectra of the PVA film and PVA/CSO nanofibers.

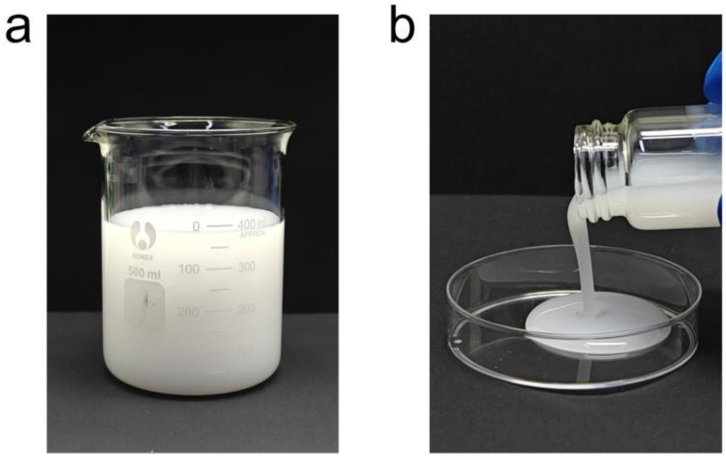

Supplementary Fig. 7. (a, b) Optical photograph of the PVA/CSO slurry, exhibiting relatively high viscosity and good fluidity.

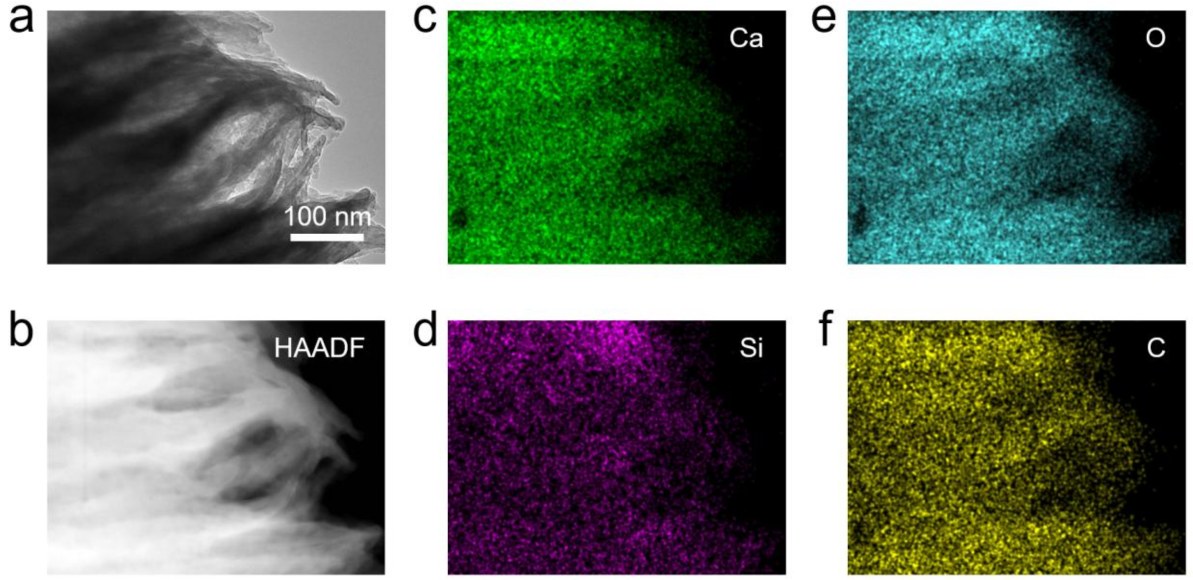

Supplementary Fig. 8. (a) TEM image of the PVA/CSO nanofiber bundles. (b) HAADF-STEM micrograph of the PVA/CSO nanofiber bundles. (c−f) Corresponding elemental mapping of Ca, Si, O, and C.

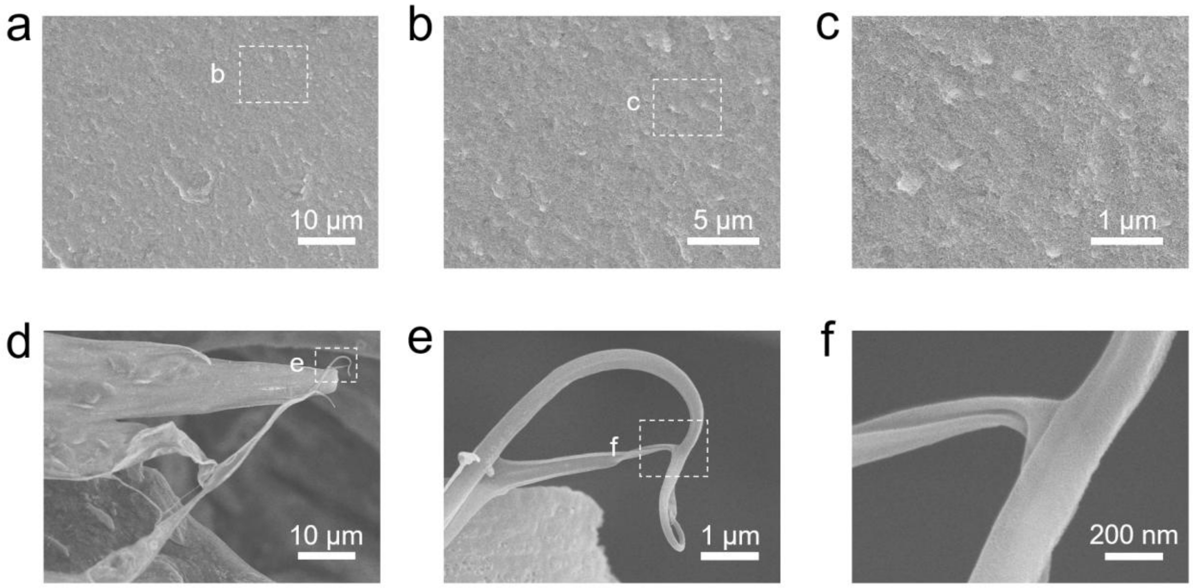

Supplementary Fig. 9. (a−c) Surface SEM images of the PVA/CSO bulk. (d−f) Cross-sectional SEM images of the PVA/CSO bulk.

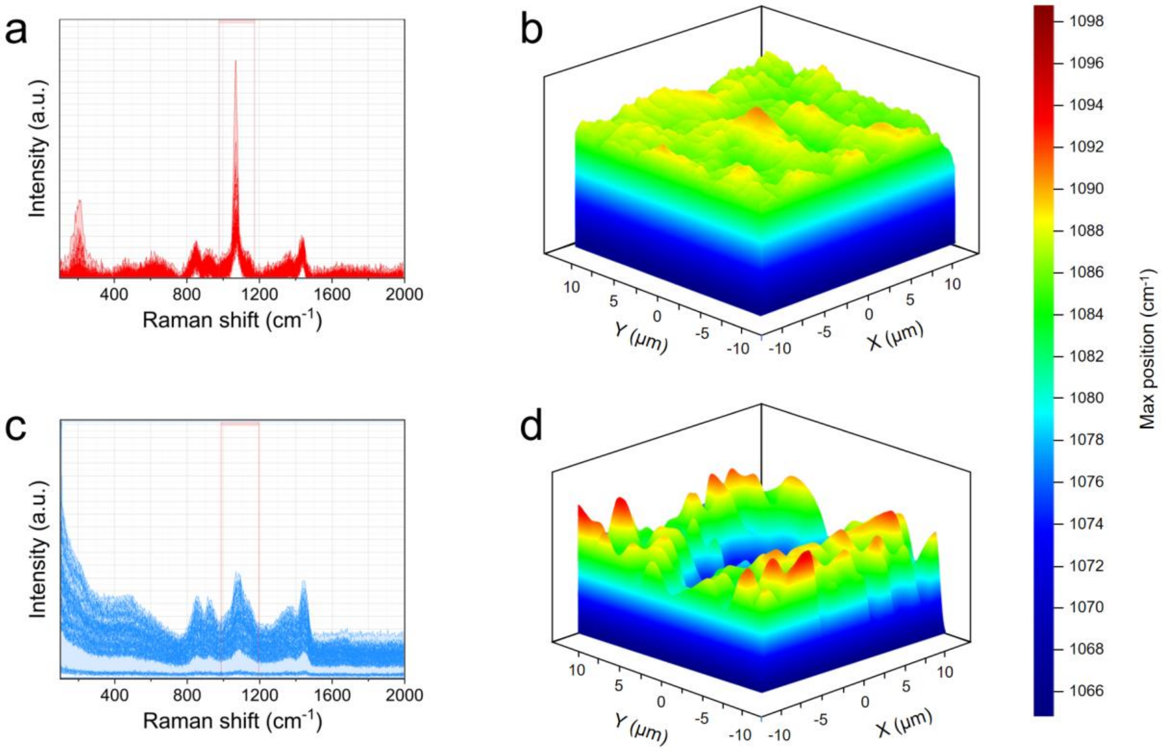

Supplementary Fig. 10. (a) 1D Raman spectra obtained by point-by-point scanning within a specific area on the surface of the PVA/CSO bulk. (b) 2D Raman mapping of Si−O peak positions on the surface of the PVA/CSO bulk. (c) 1D Raman spectra obtained by point-by-point scanning within a specific area on the surface of the PVA/CSNP bulk. (d) 2D Raman mapping of Si−O peak positions on the surface of the PVA/CSNP bulk.

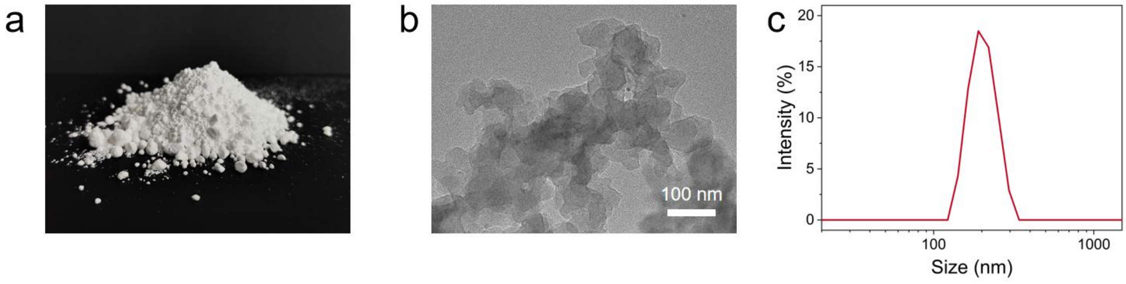

Supplementary Fig. 11. (a) Optical photograph of CaS nanoparticles. (b) TEM image of CaS nanoparticles. (d) DLS size distribution of CaS nanoparticles.

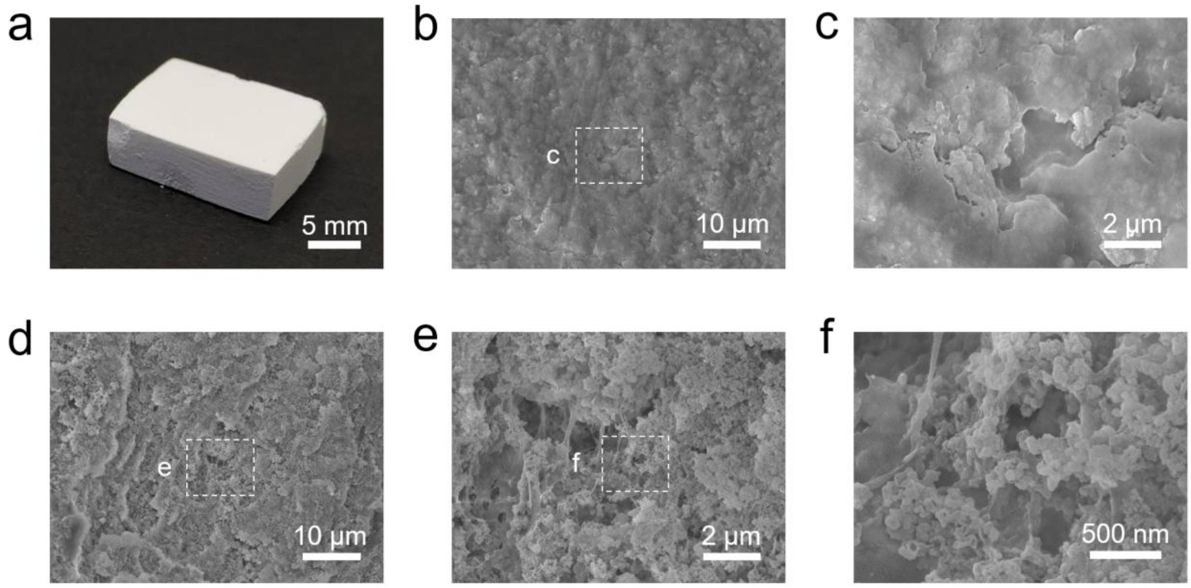

Supplementary Fig. 12. (a) Optical photograph of the PVA/CSNP bulk. (b, c) Surface SEM images of the PVA/CSNP bulk. (d–f) Cross-sectional SEM images of the PVA/CSNP bulk.

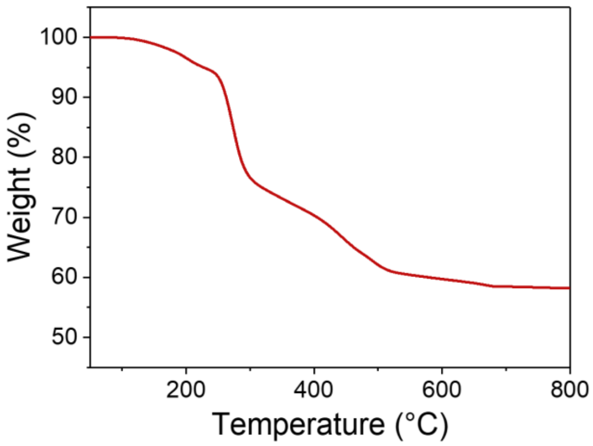

Supplementary Fig. 13. TGA pattern of the PVA/CSNP bulk.

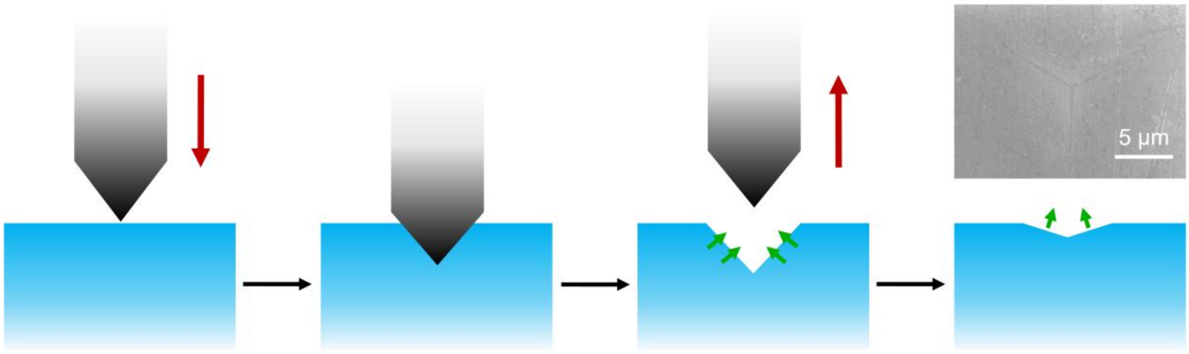

Supplementary Fig. 14. Schematic illustration of the deformation and recovery process on the surface of the PVA/CSO bulk during nanoindentation. The upper-right inset presents an SEM image of the residual indentation left on the PVA/CSO bulk surface after nanoindentation.

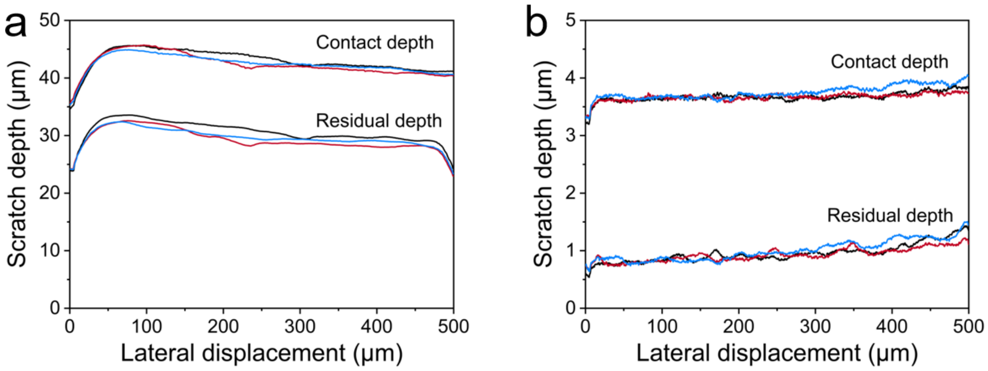

Supplementary Fig. 15. (a) Contact depth and residual depth versus lateral displacement curves for the PVA/CSNP bulk. (b) Contact depth and residual depth versus lateral displacement curves for the PVA/CSO bulk.

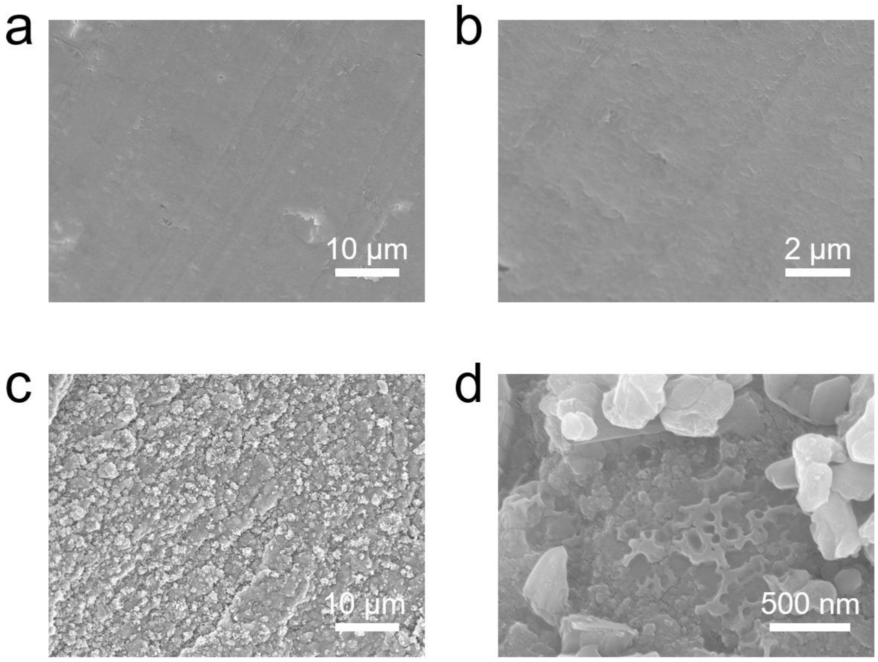

Supplementary Fig. 16. (a, b) Surface SEM images of the smart PCG elastic mineral. (c, d) Cross-sectional images of the smart PCG elastic mineral.
