# Supplementary material for: One-dimensional inorganic ionic polymerization for elastic minerals
Source: Nat Commun. 2026 May 27;17:6903. doi: 10.1038/s41467-026-72767-0 (PMC13389460; doi:10.1038/s41467-026-72767-0)
Supplement: Supplementary file 2 — Description of Additional Supplementary Files [file 41467_2026_72767_MOESM2_ESM.pdf]

Description of Additional Supplementary Files

Supplementary Movie 1: This movie illustrates the brittle fracture behavior of the PVA/CSNP bulk during a single-cycle compression process, with a maximum compressive strain of 10% and a compression rate of 2 mm min^{-1}.

Supplementary Movie 2: This movie illustrates the elastic deformation and recovery behavior of the PVA/CSO elastic mineral during a single-cycle compression process, with a maximum compressive strain of 10% and a compression rate of 2 mm min^{-1}.

Supplementary Movie 3: This movie demonstrates the behavior of the smart PCG elastic mineral as a strain sensor during a single-cycle compression process. Under compressive loading, the 3D network within the PCG undergoes elasto-plastic deformation, causing dispersed graphite particles to interconnect and form conductive pathways for electrons. This results in a significant decrease in resistance, circuit conduction, and the activation of both audible and visual alarms. Upon unloading, the 3D network recovers reversibly to its original state, the circuit is interrupted accordingly, and the alarms cease.
