# Supplementary material for: One-dimensional inorganic ionic polymerization for elastic minerals
Source: Nat Commun. 2026 May 27;17:6903. doi: 10.1038/s41467-026-72767-0 (PMC13389460; doi:10.1038/s41467-026-72767-0)
Supplement: Supplementary file 6 — Peer Review file [file 41467_2026_72767_MOESM6_ESM.pdf]

nature portfolio

# Peer Review File

## One-dimensional inorganic ionic polymerization for elastic minerals

Corresponding Author: Professor Yadong Yu

**This file contains all reviewer reports in order by version, followed by all author rebuttals in order by version.**

Version 0:

Reviewer comments:

Reviewer #1

(Remarks to the Author)
This manuscript presents a novel approach to prepare elastic minerals with a 3D crosslinked network structure by inducing 1D inorganic ionic polymerization of CSO mediated by PVA molecular chains. This work effectively integrates the high hardness and strength of inorganic minerals with the high elastic recovery properties of elastomers, resulting in the high-performance PVA/CSO elastic minerals. Furthermore, by incorporating functional graphite nanoparticles, a smart sensor capable of real-time early warning in dynamic load environments was successfully developed, thereby demonstrating promising potential for practical applications. The research design is novel; the experimental data are robust; and the characterization methods are comprehensive, thereby representing significant progress in addressing the inherent brittleness of traditional inorganic ionic minerals. The proposed 1D inorganic ionic polymerization strategy is conceptually clear and opens a new pathway for the design and preparation of high-performance elastic minerals.
Overall, this work represents a significant advancement in material design, exhibiting high originality and broad interdisciplinary impact, and aligns well with the publication criteria of Nature Communications in terms of novelty, significance, and broad interest. Therefore, I strongly recommend its publication in Nature Communications. However, prior to final acceptance, the authors should consider addressing and refining the following aspects.

1) Compared to previously reported calcium carbonate and calcium phosphate oligomers, the silicate ions used in the preparation of CSO are highly unstable and prone to condensation into network structures. However, the synthesis approach developed in this study effectively suppresses silicate condensation, successfully producing oligomers with an average particle size of only 1.5 nm. It is recommended to further elaborate on the underlying stabilization mechanism.
2) There are minor formatting errors in the text. For example, the authors frequently refer to “ionic-molecular chains”, but there is inconsistent use of hyphens and en dashes. It is recommended to review and standardize the usage of symbols throughout the manuscript. And I suggest to change “1D” to “One-dimensional” in title.
3) The authors employed 1D SAXS to analyze the shape and size distribution of CSO in detail. However, corresponding characterization of the ionic-molecular chains in PVA/CSO is lacking. It is recommended to supplement this characterization and provide a comparative explanation of the differences between the two, thereby making the structural characterization of 1D ionic-molecular chains more comprehensive.
4) The authors note that the ethanol dispersion of CSO exhibits stable dispersion and suspension properties. Providing details on the stable storage duration under standard temperature conditions would further enhance the reproducibility of future studies.
5) The authors should specify the precise graphite content in the PCG smart elastic mineral (expressed as a mass percentage). This information should be explicitly stated. Additionally, a concise analysis of how excessive or insufficient graphite content affects the conductivity, mechanical properties, and sensing stability of the PCG material would provide readers with a clearer understanding of the composition–performance relationship of the composite.
6) In this study, the authors developed a PCG conductive elastic mineral for sensing applications, such as earthquake and bridge overload warnings. This is an innovative and novel design. It would be valuable to clarify whether this material could be adapted for other practical scenarios.

Reviewer #2

(Remarks to the Author)

Du et al. guided the 1D inorganic ionic polymerization of CSO using PVA molecular chains, forming flexible PVA/CSO composite chains rich in ionic defects, and constructed an elastomer-like 3D crosslinked network through hierarchical assembly. This design effectively integrates the high strength of inorganic ionic minerals with the high elasticity of organic elastomers. The prepared PVA/CSO material not only maintains high hardness (~0.78 GPa), high modulus (~20.63 GPa), and high specific strength (~74.53 MPa $g^{-1}$ $cm^3$), but also exhibits compressive plastic deformation exceeding 50%. Furthermore, it demonstrates complete recovery after 5,000 cycles at 10% strain, highlighting exceptional elasticity and fatigue resistance. Additionally, the PCG smart material, prepared by incorporating graphite, successfully achieves strain sensing and real-time early warning capabilities. The core innovation of this work lies in proposing a novel "1D inorganic ionic polymerization" strategy and successfully constructing hybrid materials that combine the strength of minerals with the elasticity of elastomers, representing a significant breakthrough in material design philosophy. The experimental data are comprehensive, the characterization is thorough, and the conclusions are reliable. It is recommended for acceptance after the following revisions are addressed.

1. The preparation and selection of CSO are crucial to the performance of elastic mineral materials. The authors are advised to provide additional justification for the use of a TEA ratio of 1:20 during the CSO preparation process and to explain the rationale for centrifuging the CSO dispersion at 6,000 rpm to obtain the precipitate before compounding it with the PVA solution.
2. In Fig. 1h, the scale bar on the far right overlaps with the dashed line. It is recommended to adjust the relative position to improve visual clarity.
3. Key details in the preparation process of the elastic mineral materials are described somewhat vaguely, such as the "loss of fluidity and transition into a gel state" and the deformation state induced by prestress in Fig. 4. It is recommended to supplement critical parameters, such as water content, for clearer definition.
4. To enhance the clarity and consistency of the graph, a scale bar should be added to the image in Fig. 2h.
5. The authors should verify whether the magnification levels of the SEM images in Fig. 3e are consistent. If they are not, a corresponding scale bar should be added to the PVA/CSNP image section.
6. Microscopic morphology and mechanical testing are often related to sample composition and inorganic content. The authors should clarify whether the organic–inorganic composition ratios of the PVA/CSO and PVA/CSNP samples used in the experiments were consistent.
7. The authors note that the introduction of graphite particles leads to hysteresis loops in the stress–strain curve of PCG, indicating some influence on its elastic network, with an elastic recovery rate of approximately 68.4% (Fig. 4h). How was this elastic recovery rate calculated?

Version 1:

Reviewer comments:

Reviewer #1

(Remarks to the Author)
The author had addressed most of my comments. I have one comment to clarify: In Comment-5, the XRD can reflect whether the sample crystallized. However, calcium silicate solid prefers amorphous phase for most cases, this means the XRD pattern would not change obviously. In contrast, the stable dispersion and suspension property can be presented by a snapshot of entire sample. For example, no precipitation of phase separation appears for CSO.

Reviewer #2

(Remarks to the Author)
The authors supplemented the necessary characterization experiments, description of the experimental procedure, and analysis of the experimental results, and all concerns have been addressed.I recommend acceptance of the manuscript for publication.

Version 2:

Reviewer comments:

Reviewer #1

(Remarks to the Author)
The authors have well addressed my comment.

**Open Access** This Peer Review File is licensed under a Creative Commons Attribution 4.0 International License, which permits use, sharing, adaptation, distribution and reproduction in any medium or format, as long as you give appropriate credit to the original author(s) and the source, provide a link to the Creative Commons license, and indicate if changes were made.
In cases where reviewers are anonymous, credit should be given to 'Anonymous Referee' and the source.
The images or other third party material in this Peer Review File are included in the article's Creative Commons license, unless indicated otherwise in a credit line to the material. If material is not included in the article's Creative Commons license and your intended use is not permitted by statutory regulation or exceeds the permitted use, you will need to obtain permission directly from the copyright holder.
To view a copy of this license, visit https://creativecommons.org/licenses/by/4.0/

# Point-by-Point Response

NCOMMS-26-004825-T, "1D inorganic ionic polymerization for elastic minerals"

We thank all referees for their comments and their constructive criticisms are helpful to improve our work. As per the suggestions, we have carefully improved the manuscript and all changes are highlighted by yellow in the revised manuscript and Supporting Information. The point-by-point responses are also listed as follows:

## Referee #1

**Comment-1:** *This manuscript presents a novel approach to prepare elastic minerals with a 3D crosslinked network structure by inducing 1D inorganic ionic polymerization of CSO mediated by PVA molecular chains. This work effectively integrates the high hardness and strength of inorganic minerals with the high elastic recovery properties of elastomers, resulting in the high-performance PVA/CSO elastic minerals. Furthermore, by incorporating functional graphite nanoparticles, a smart sensor capable of real-time early warning in dynamic load environments was successfully developed, thereby demonstrating promising potential for practical applications. The research design is novel; the experimental data are robust; and the characterization methods are comprehensive, thereby representing significant progress in addressing the inherent brittleness of traditional inorganic ionic minerals. The proposed 1D inorganic ionic polymerization strategy is conceptually clear and opens a new pathway for the design and preparation of high-performance elastic minerals.*

*Overall, this work represents a significant advancement in material design, exhibiting high originality and broad interdisciplinary impact, and aligns well with the publication criteria of Nature Communications in terms of novelty, significance, and broad interest. Therefore, I strongly recommend its publication in Nature Communications. However, prior to final acceptance, the authors should consider addressing and refining the following aspects.*

**Response:** Thanks for the positive response and constructive suggestions. As per the comments, we have made related changes in the revised manuscript. Please see more details below.

**Comment-2:** *Compared to previously reported calcium carbonate and calcium phosphate oligomers, the silicate ions used in the preparation of CSO are highly unstable and prone to condensation into network structures. However, the synthesis approach developed in this study effectively suppresses silicate condensation, successfully producing oligomers with an average particle size of only 1.5 nm. It is recommended to further elaborate on the underlying stabilization mechanism.*

**Response:** Thanks for the comment. In the synthesis of CSO, the stabilization mechanism that inhibits the condensation of silicate ions primarily involves the following aspects: First, during the

synthesis of CSO, ethanol was selected as the solvent. As shown in Supplementary Fig. 1c, after centrifugation at 8000 rpm for 5 min, the CSO dispersion formed a transparent gel containing the solvent, indicating strong intermolecular interactions between calcium silicate ions and ethanol molecules. This facilitates efficient solvation of silicate ions, significantly reducing the hydrolysis and condensation rates of silicate species. Second, the nitrogen atom in TEA possesses lone pair electrons, enabling it to form strong hydrogen bonds with silanol groups (Si–OH). As illustrated in Supplementary Fig. 1d, e, after the formation of CSO, the stretching vibration peaks of the C–N bond in TEA exhibited shifts (from 1200 $cm^{-1}$ to 1203 $cm^{-1}$ in ATR-FTIR and from 735.5 $cm^{-1}$ to 739 $cm^{-1}$ in Raman spectroscopy), confirming that TEA binds to silanol groups through hydrogen bonding. This hydrogen bonding interaction not only reduces the condensation reactivity of silanol groups but also forms a charge protection layer on the surface of CSO, enhancing the repulsive barrier between CSO particles, thereby kinetically hindering their collision, aggregation, and further condensation.

To address this comment, we have added more detailed explanations in the revised manuscript, and they now read: "Concurrently, ethanol as a solvent significantly slows down the hydrolysis and condensation rates of silicates, effectively inhibiting the condensation of silicate ions." and "This hydrogen bonding interaction not only reduces the condensation reactivity of silanol groups (Si–OH) but also forms a charge protection layer on the surface of CSO, enhancing the repulsive barrier between CSO particles, thereby kinetically hindering their collision, aggregation, and further condensation." on Page 5.

**Comment-3:** *There are minor formatting errors in the text. For example, the authors frequently refer to "ionic-molecular chains", but there is inconsistent use of hyphens and en dashes. It is recommended to review and standardize the usage of symbols throughout the manuscript. And I suggest to change "1D" to "One-dimensional" in title.*

**Response:** Thanks for the careful reading. We have thoroughly reviewed and revised the entire manuscript to ensure the standardized use of hyphens and en dashes. Additionally, following your suggestion, "1D" in the title has been revised to "One-dimensional". The title of the manuscript now reads: "One-dimensional inorganic ionic polymerization for elastic minerals" on Page 1.

**Comment-4:** *The authors employed 1D SAXS to analyze the shape and size distribution of CSO in detail. However, corresponding characterization of the ionic-molecular chains in PVA/CSO is lacking. It is recommended to supplement this characterization and provide a comparative explanation of the differences between the two, thereby making the structural characterization of 1D ionic-molecular chains more comprehensive.*

**Response:** Thanks for the comment. We have supplemented 1D SAXS analyze of the ionic-molecular chains in the PVA/CSO dispersion (result shown in Supplementary Fig. 4 below). The results confirmed the presence of nanofibers with an average width of 9.1 nm in the PVA/CSO dispersion (Supplementary Fig. 4), which is consistent with the TEM observations. This further indicates that, through the induction of PVA molecular chains, CSO gradually undergoes oriented

polymerization to form PVA/CSO ionic-molecular chains, which subsequently assemble into nanofibers.

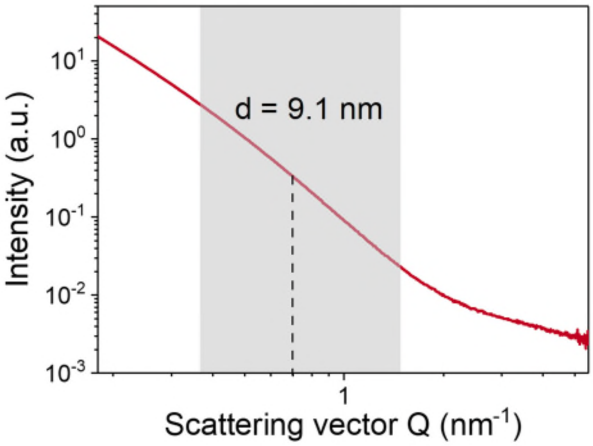


**Supplementary Fig. 4**. 1D SAXS pattern of the ionic-molecular chains in the PVA/CSO dispersion.

To address this comment, we have added the corresponding figure in the revised Supporting Information and added corresponding explanations in the main text, and now it reads: "Furthermore, the 1D SAXS pattern confirmed the presence of nanofibers with an average width of 9.1 nm within the PVA/CSO system (Supplementary Fig. 4), which is consistent with the TEM observations. This finding further demonstrates that, facilitated by the PVA molecular chains, CSO undergo oriented polymerization, forming PVA/CSO ionic-molecular chains that self-assemble into nanofibers." on Page 8.

**Comment-5:** *The authors note that the ethanol dispersion of CSO exhibits stable dispersion and suspension properties. Providing details on the stable storage duration under standard temperature conditions would further enhance the reproducibility of future studies.*

**Response:** Thanks for the comment. To investigate the stability of CSO, a CSO dispersion prepared approximately 6 months prior was centrifuged, washed, and dried to obtain a powdered sample, followed by XRD characterization (result shown in Supplementary Fig. 2). The experimental data indicate that, similar to the previously synthesized calcium phosphate oligomers, CSO remains in an amorphous state after 6 months of storage, confirming its excellent long-term stability of at least 6 months.

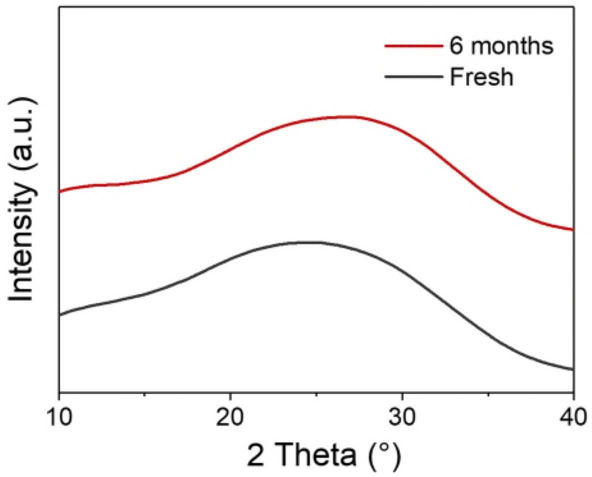


**Supplementary Fig. 2.** XRD pattern of freshly prepared CSO and CSO after approximately 6 months of storage.

To address this comment, we have added the corresponding figure in the revised Supporting Information and added corresponding explanations in the main text, and now it reads: "Furthermore, the XRD pattern of the as-prepared CSO remained amorphous after 6 months of storage (Supplementary Fig. 2), indicating that the CSO synthesized using this strategy exhibit good long-term stability." on Page 7.

**Comment-6:** *The authors should specify the precise graphite content in the PCG smart elastic mineral (expressed as a mass percentage). This information should be explicitly stated. Additionally, a concise analysis of how excessive or insufficient graphite content affects the conductivity, mechanical properties, and sensing stability of the PCG material would provide readers with a clearer understanding of the composition–performance relationship of the composite.*

**Response:** Thanks for the comment. In this study, the mass percentage of graphite in the PCG smart elastic mineral is approximately 10.0 wt%. Indeed, the graphite content is critical to the performance of PCG. When the content is significantly lower than 10.0 wt%, the performance of PCG shows no notable difference from that of the PVA/CSO elastic mineral. Specifically, the graphite particles fail to form continuous conductive pathways, rendering the material insulating or exhibiting extremely high resistance, which makes it incapable of sensing functionality. When the content is moderate, the electrical resistance becomes sensitive to strain or stress, as illustrated in Fig. 4j, k. When the content is excessive, the conductive network becomes overly dense, maintaining good conductivity even in the unstrained state, leading to a narrow resistance variation range and reduced sensitivity. Moreover, excessive graphite may aggregate, disrupting the integrity of the PVA/CSO ionic-molecular chain crosslinking network. This creates stress concentration points, induces microcracks, and reduces the elastic recovery rate, making the material brittle. Once permanent deformation occurs, the sensing stability and accuracy are severely compromised. Therefore, only with a moderate graphite content (such as the 10 wt% selected in this study) can the material achieve high sensitivity, a broad sensing range, and excellent cyclic stability while preserving good elasticity and strength.

To address this comment, we have added a more detailed explanation in the revised manuscript, and it now reads: "It is noteworthy that the graphite content exerts a decisive influence on the sensing performance of PCG. In this study, the graphite content in PCG was optimized to 10.0 wt%. When the graphite content is too low, the particles fail to form a continuous conductive network, rendering PCG electrically insulating or characterized by excessively high resistance, thereby preventing effective sensing. Conversely, when the graphite content is excessive, the conductive network becomes overly dense, enabling PCG to maintain high conductivity even in the unstrained state, which results in a limited resistance variation range and reduced sensitivity. More critically, excess graphite tends to aggregate, disrupting the integrity of the PVA/CSO ionic–molecular crosslinked network, generating stress concentration sites, inducing microcracks, and reducing the elastic recovery capability of the material, thereby increasing brittleness. Once irreversible deformation occurs, the stability and accuracy of the sensor are significantly compromised. Therefore, an optimal graphite content of 10.0 wt% enables PCG to simultaneously achieve excellent mechanical strength, high sensitivity, a wide sensing range, and outstanding cyclic stability." on Page 21.

**Comment-7:** *In this study, the authors developed a PCG conductive elastic mineral for sensing applications, such as earthquake and bridge overload warnings. This is an innovative and novel design. It would be valuable to clarify whether this material could be adapted for other practical scenarios.*

**Response:** Thanks for the comment. Owing to its excellent elasticity, high hardness, good machinability, and reversible strain-response characteristics, the PCG intelligent elastic mineral material exhibits significant potential for applications involving the monitoring of deformation, pressure, or vibration. For example, it can be employed to monitor strain, vibration, and fatigue damage in large-scale mechanical equipment (e.g., wind turbine blades and turbines), as well as in pipelines, pressure vessels, and aerospace structural components, thereby enabling predictive maintenance. When integrated into robotic grippers, it can function as a tactile sensor for intelligent robots, enabling perception of gripping force and object morphology, thereby enhancing operational dexterity and interaction safety.

In summary, the core advantage lies in integrating the high strength and wear resistance of traditional mineral materials with the reversible deformation capability of polymers while simultaneously imparting electrical sensing functionality. This integration enables long-term and stable strain perception in harsh mechanical environments requiring high wear resistance and load-bearing capacity, which are capabilities that many purely polymer-based flexible sensors struggle to achieve. Therefore, the PCG intelligent elastic mineral material demonstrates broad application prospects in smart structural systems that require the integration of load-bearing and condition-sensing functions.

To address this comment, we have added a more detailed explanation in the revised manuscript, and it now reads: "More importantly, PCG integrates the high strength and wear resistance of mineral materials, the reversible deformation capability of polymer elastomers, and electrical sensing functionality into a single material system, thereby achieving multifunctional integration of

load-bearing and sensing. This characteristic enables long-term, stable strain perception in harsh service environments characterized by high wear and heavy loads, thereby overcoming the limitations of conventional purely polymer-based flexible sensors in terms of mechanical durability and load-bearing capacity. Leveraging this advantage, PCG demonstrates broad application prospects in smart structures and intelligent equipment. It can be employed not only for health monitoring and predictive maintenance of critical large-scale equipment, such as wind turbine blades, turbines, pipeline systems, and aerospace structural components, but also integrated into robotic grippers to provide high-precision tactile perception, thereby significantly enhancing operational intelligence and safety." on Page 20.

## Referee #2

**Comment-1:** *Du et al. guided the 1D inorganic ionic polymerization of CSO using PVA molecular chains, forming flexible PVA/CSO composite chains rich in ionic defects, and constructed an elastomer-like 3D crosslinked network through hierarchical assembly. This design effectively integrates the high strength of inorganic ionic minerals with the high elasticity of organic elastomers. The prepared PVA/CSO material not only maintains high hardness (~0.78 GPa), high modulus (~20.63 GPa), and high specific strength (~74.53 MPa $g^{-1}$ $cm^3$), but also exhibits compressive plastic deformation exceeding 50%. Furthermore, it demonstrates complete recovery after 5,000 cycles at 10% strain, highlighting exceptional elasticity and fatigue resistance. Additionally, the PCG smart material, prepared by incorporating graphite, successfully achieves strain sensing and real-time early warning capabilities. The core innovation of this work lies in proposing a novel "1D inorganic ionic polymerization" strategy and successfully constructing hybrid materials that combine the strength of minerals with the elasticity of elastomers, representing a significant breakthrough in material design philosophy. The experimental data are comprehensive, the characterization is thorough, and the conclusions are reliable. It is recommended for acceptance after the following revisions are addressed.*

**Response:** Thanks for the positive response and constructive suggestions. As per the comments, we have made related changes in the revised manuscript. Please see more details below.

**Comment-2:** *The preparation and selection of CSO are crucial to the performance of elastic mineral materials. The authors are advised to provide additional justification for the use of a TEA ratio of 1:20 during the CSO preparation process and to explain the rationale for centrifuging the CSO dispersion at 6,000 rpm to obtain the precipitate before compounding it with the PVA solution.*

**Response:** Thanks for the comment. The specific selection of a 1:20 TEA-to-calcium ion molar ratio and a centrifugation speed of 6000 rpm for separating CSO during the preparation process was determined based on prior experimental experience. Specifically, the 1:20 ratio represents an optimized stoichiometry. TEA exerts its capping effect by forming hydrogen bonds with silanol groups on the surface of CSO precursors, thereby preventing excessive growth of inorganic ions and

avoiding the formation of three-dimensional networks such as silica gels. If the TEA content is too low, some active sites may remain uncapped, leading to partial condensation, particle enlargement, and a broader particle size distribution. Conversely, excess TEA alters the pH and ionic strength of the reaction system, thereby catalyzing undesirable hydrolysis and silicate condensation reactions. Therefore, the 1:20 ratio achieves an optimal balance between effective capping and minimal interference with the reaction system.

The centrifugation speed of 6000 rpm for separating CSO was selected to balance centrifugal yield with the efficiency of subsequent synthesis processes. Following the CSO synthesis reaction, the system contains unreacted precursors along with CSO of approximately 1.5 nm in size. This centrifugal force is sufficient to sediment the target-sized CSO while retaining smaller, more soluble species in the supernatant. If the speed is too low, the CSO may not be fully precipitated, resulting in reduced yield. Conversely, if the speed is too high, the precipitate may become excessively compacted, hindering its redispersion in the PVA solution.

Therefore, the 1:20 CSO-to-TEA ratio and a centrifugation rate of 6000 rpm were selected for this study.

To address this comment, we have added the corresponding explanations in the main text, and they now read: "Specifically, the hydrolysis of tetraethyl orthosilicate (TEOS) was carefully controlled to release silicate ions, which subsequently reacted with calcium ions in ethanol. Triethylamine (TEA) was employed as a capping agent, with the calcium-to-TEA molar ratio fixed at 1:20, to synthesize CSO. This ratio ensures effective surface capping while preventing particle enlargement caused by insufficient TEA and minimizing undesirable side reactions induced by excess TEA." on Page 4 and "This centrifugation speed effectively precipitates CSO of approximately 1.5 nm while retaining smaller soluble species in the supernatant, thereby avoiding yield loss from excessively low speeds and redispersion difficulties caused by overly compacted precipitates at excessively high speeds." on Page 23.

**Comment-3:** *In Fig. 1h, the scale bar on the far right overlaps with the dashed line. It is recommended to adjust the relative position to improve visual clarity.*

**Response:** Thanks for the comment. As per the suggestion, we have adjusted the position of the scale bar in Fig. 1h in the revised manuscript. The updated Fig. 1h is shown below.

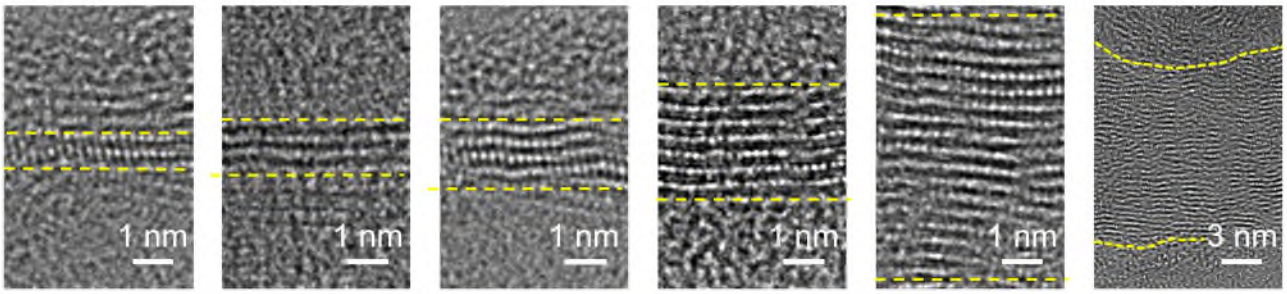


**Fig. 1h**

**Comment-4:** *Key details in the preparation process of the elastic mineral materials are described somewhat vaguely, such as the "loss of fluidity and transition into a gel state" and the deformation state induced by prestress in Fig. 4. It is recommended to supplement critical parameters, such as water content, for clearer definition.*

**Response:** Thanks for the comment. To provide a more precise definition of the "loss of fluidity and transition into a gel state" and the "deformation state induced by prestress in Fig. 4", we have added detailed explanations in the revised manuscript, and they now read: "Furthermore, by applying pre-stress in different directions to the initially formed PVA/CSO gel sheets (with a water content of approximately 25%) during the drying process, complex centimeter-scale curled sheet structure can be produced (Fig. 4d), fully illustrating their exceptional plasticity and processing adaptability." on Page 17~18, "The slurry was then transferred to a culture dish and dried at 25 °C until it lost fluidity and transitioned into a gel state, thus yielding the PVA/CSO precursor gel with a water content of about 25%." and "This slurry was subsequently transferred to a culture dish and dried at 25 °C until it ceased flowing and gelled, thereby yielding the PCG precursor gel with a water content of about 25%." on Page 23.

**Comment-5:** *To enhance the clarity and consistency of the graph, a scale bar should be added to the image in Fig. 2h.*

**Response:** Thanks for the comment. As per the suggestion, we have added a scale bar to Fig. 2h in the revised manuscript, and the updated figure is shown below.

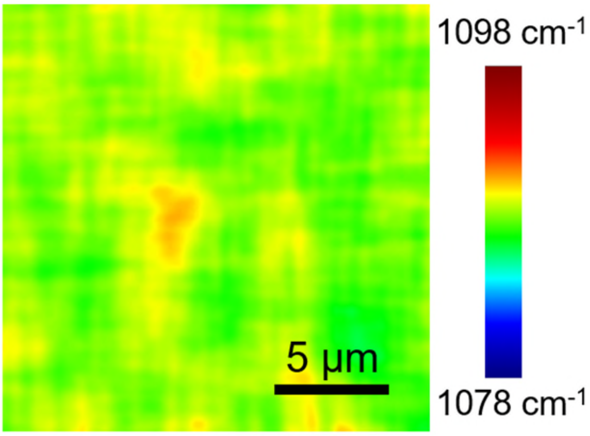


**Fig. 2h**

**Comment-6:** *The authors should verify whether the magnification levels of the SEM images in Fig. 3e are consistent. If they are not, a corresponding scale bar should be added to the PVA/CSNP image section.*

**Response:** Thanks for the comment. The magnification of the SEM images in Fig. 3e is consistent, and we have added a scale bar to the PVA/CSNP group in the revised manuscript. The updated

figure is shown below. Additionally, we have carefully examined all other SEM and TEM images to ensure the accuracy of the scale bars.

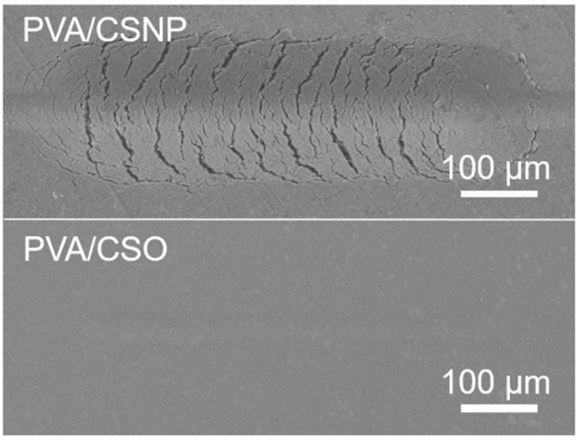


**Fig. 3e**

**Comment-7:** *Microscopic morphology and mechanical testing are often related to sample composition and inorganic content. The authors should clarify whether the organic–inorganic composition ratios of the PVA/CSO and PVA/CSNP samples used in the experiments were consistent.*

**Response:** Thanks for the comment. In this study, we first obtained PVA/CSO elastic mineral with an inorganic content of 58.1 wt% (Supplementary Fig. 3c). To further verify the uniqueness of CSO in constructing elastic minerals, we simultaneously prepared PVA/CSNP bulk using calcium silicate nanoparticles and ensured that its inorganic content was consistent with that of the PVA/CSO elastic mineral. To this end, we determined the inorganic content of the obtained PVA/CSNP bulk via TGA, with the results shown in Supplementary Fig. 13. The test result indicates that the inorganic component content in PVA/CSNP is approximately 58.2 wt%, confirming that the organic–inorganic composition ratio of the PVA/CSNP bulk is essentially consistent with that of the PVA/CSO elastic mineral.

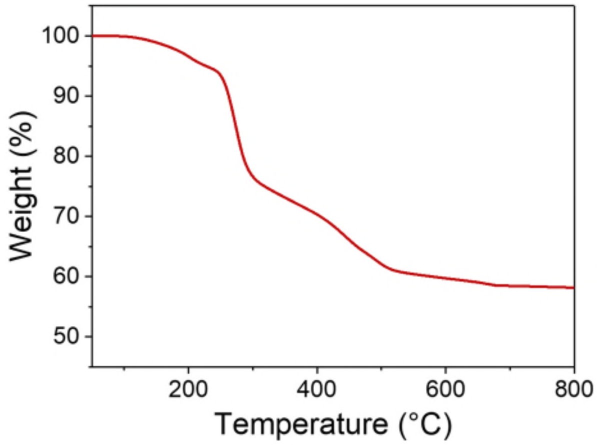


**Supplementary Fig. 13.** TGA pattern of the PVA/CSNP bulk.

To address this comment, we have added the corresponding figure in the revised Supporting Information and added corresponding explanations in the main text, and now it reads: "The resulting PVA/CSNP bulk exhibited an organic–inorganic composition ratio consistent with that of the PVA/CSO bulk (Supplementary Fig. 13)." on Page 12~13.

**Comment-8:** *The authors note that the introduction of graphite particles leads to hysteresis loops in the stress–strain curve of PCG, indicating some influence on its elastic network, with an elastic recovery rate of approximately 68.4% (Fig. 4h). How was this elastic recovery rate calculated?*

**Response:** Thanks for the comment. The elastic recovery rate was calculated from cyclic loading–unloading curves. For each loading–unloading cycle, the maximum applied strain ($\varepsilon_{max}$, which is 10% in this case) and the residual strain upon unloading when the stress returns to zero ($\varepsilon_{res}$) were recorded. The elastic recovery rate ($R_\varepsilon$) was calculated using the following equation:

$$R_\varepsilon = (\varepsilon_{max} - \varepsilon_{res})/\varepsilon_{max} \times 100\%$$

To address this comment, we have added a more detailed explanation in the revised manuscript, and it now reads: "The elastic recovery rate was calculated based on the cyclic loading–unloading curves. For each loading–unloading cycle, the maximum applied strain ($\varepsilon_{max}$, which is 10% in this case) and the residual strain upon unloading when the stress returns to zero ($\varepsilon_{res}$) were recorded. The elastic recovery rate ($R_\varepsilon$) was calculated using the following equation:

$$R_\varepsilon = (\varepsilon_{max} - \varepsilon_{res})/\varepsilon_{max} \times 100\% \qquad (2)$$"

on Page 26.

# Point-by-Point Response

NCOMMS-26-004825A, "One-dimensional inorganic ionic polymerization for elastic minerals"

We thank all referees for their comments and their constructive criticisms are helpful to improve our work. As per the suggestions, we have carefully improved the manuscript and all changes are highlighted by yellow in the Revised Manuscript and Supporting Information. The point-by-point responses are also listed as follows:

## Referee #1

**Comment-1:** *The author had addressed most of my comments. I have one comment to clarify: In Comment-5, the XRD can reflect whether the sample crystallized. However, calcium silicate solid prefers amorphous phase for most cases, this means the XRD pattern would not change obviously. In contrast, the stable dispersion and suspension property can be presented by a snapshot of entire sample. For example, no precipitation of phase separation appears for CSO.*

**Response:** We greatly appreciate your further guidance and valuable comments on our work. We are pleased that most of the comments have been appropriately addressed, and we also thank you for the detailed explanation regarding Comment-5. Indeed, XRD analysis can only confirm that the CSO is in an amorphous state and cannot directly reflect its dispersion stability.

As per suggestion, we re-prepared fresh CSO, which were washed with ethanol and dispersed in ethanol to maintain a concentration of 20 mg $mL^{-1}$. These were then compared with CSO dispersions (concentration 20 mg $mL^{-1}$) that had been stored in the laboratory for approximately 6 months. The results show that the CSO dispersion stored for about 6 months (Supplementary Fig. 2b) is essentially identical in appearance to the freshly prepared CSO dispersion (Supplementary Fig. 2a). More importantly, the CSO dispersion stored for 6 months still exhibited excellent uniform suspendability and fluidity, with no signs of sedimentation, aggregation, or phase separation (Supplementary Fig. 2c). Combined with the XRD analysis results (Supplementary Fig. 2d), which show it remains in an amorphous state with no phase transition, this fully demonstrates that CSO possesses excellent long-term dispersion stability, with a stable period of at least 6 months.

To address the comment, we have added the corresponding figure (Supplementary Fig. 2) to the revised Supporting Information and included a relevant explanation in the main text, and it now reads: "Furthermore, the as-prepared CSO dispersion, after static storage for approximately 6 months, still exhibited excellent uniform suspendability and fluidity, maintaining an appearance consistent with that of freshly prepared CSO dispersion, without any sedimentation, aggregation, or phase separation (Supplementary Fig. 2a–c). Its XRD pattern remained amorphous (Supplementary Fig. 2d). These results indicate that the CSO synthesized using this strategy possesses good long-term stability." on Page 7~8.

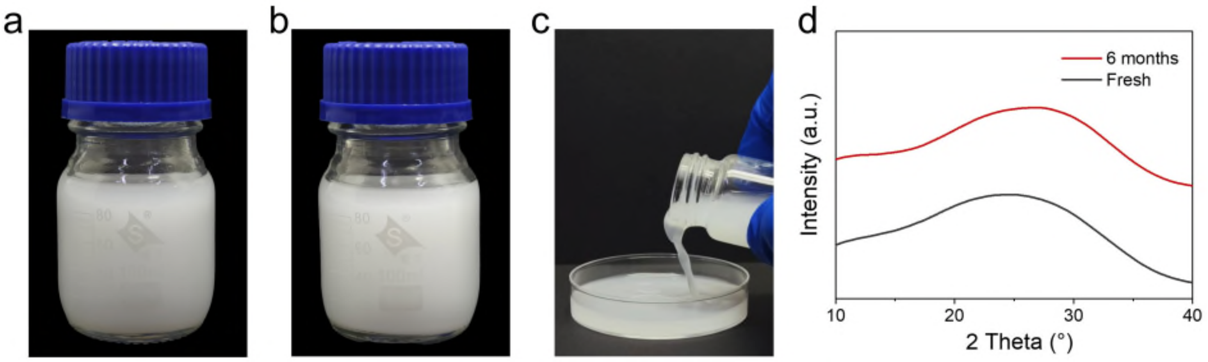


**Supplementary Fig. 2.** (a, b) Optical photographs of CSO dispersed in ethanol (20 mg $mL^{-1}$): (a) freshly prepared and (b) after approximately 6 months of storage. (c) Optical photograph of the CSO dispersion after approximately 6 months of storage, showing good fluidity. (d) XRD pattern of freshly prepared CSO and CSO after approximately 6 months of storage.

**<u>Referee #2</u>**

**Comment-1:** *The authors supplemented the necessary characterization experiments, description of the experimental procedure, and analysis of the experimental results, and all concerns have been addressed. I recommend acceptance of the manuscript for publication.*

**Response:** Thank you very much for your positive feedback and for recommending our manuscript for acceptance. We are glad that the additional experiments and revisions have fully addressed your concerns. We greatly appreciate your time and effort in reviewing our work.
